# Genomic comparison between *Staphylococcus aureus* GN strains clinically isolated from a familial infection case: IS*1272* transposition through a novel inverted repeat-replacing mechanism

**DOI:** 10.1371/journal.pone.0187288

**Published:** 2017-11-08

**Authors:** Tsai-Wen Wan, Wataru Higuchi, Olga E. Khokhlova, Wei-Chun Hung, Yasuhisa Iwao, Masataka Wakayama, Noriyoshi Inomata, Tomomi Takano, Yu-Tzu Lin, Olga V. Peryanova, Kenji K. Kojima, Alla B. Salmina, Lee-Jene Teng, Tatsuo Yamamoto

**Affiliations:** 1 Department of Epidemiology, Genomics, and Evolution, International Medical Education and Research Center, Niigata, Japan; 2 Department of Clinical Laboratory Sciences and Medical Biotechnology, National Taiwan University College of Medicine, Taipei, Taiwan; 3 Division of Bacteriology, Department of Infectious Disease Control and International Medicine, Niigata University Graduate School of Medical and Dental Sciences, Niigata, Japan; 4 Russia-Japan Center of Microbiology, Metagenomics and Infectious Diseases, Krasnoyarsk State Medical University named after Prof. V.F. Voino-Yasenetsky, Krasnoyarsk, Russia; 5 Department of Microbiology and Immunology, Kaohsiung Medical University, Kaohsiung, Taiwan; 6 Division of Clinical Laboratory, Kido Hospital, Niigata, Japan; 7 Division of Dermatology, Kido Hospital, Niigata, Japan; 8 Department of Life Science, National Cheng Kung University, Tainan, Taiwan; 9 Genetic Information Research Institute (GIRI), Mountain View, CA, United States of America; 10 Research Institute of Molecular Medicine and Pathobiochemistry, Krasnoyarsk State Medical University named after Professor V.F. Vojno-Yasenetsky, Krasnoyarsk, Russia; Pusan National University, REPUBLIC OF KOREA

## Abstract

A bacterial insertion sequence (IS) is a mobile DNA sequence carrying only the transposase gene (*tnp*) that acts as a mutator to disrupt genes, alter gene expressions, and cause genomic rearrangements. “Canonical” ISs have historically been characterized by their terminal inverted repeats (IRs), which may form a stem-loop structure, and duplications of a short (non-IR) target sequence at both ends, called target site duplications (TSDs). The IS distributions and virulence potentials of *Staphylococcus aureus* genomes in familial infection cases are unclear. Here, we determined the complete circular genome sequences of familial strains from a Panton-Valentine leukocidin (PVL)-positive ST50/*agr*4 *S*. *aureus* (GN) infection of a 4-year old boy with skin abscesses. The genomes of the patient strain (GN1) and parent strain (GN3) were rich for “canonical” IS*1272* with terminal IRs, both having 13 commonly-existing copies (ce-IS*1272*). Moreover, GN1 had a newly-inserted IS*1272* (ni-IS*1272*) on the PVL-converting prophage, while GN3 had two copies of ni-IS*1272* within the DNA helicase gene and near *rot*. The GN3 genome also had a small deletion. The targets of ni-IS*1272* transposition were IR structures, in contrast with previous “canonical” ISs. There were no TSDs. Based on a database search, the targets for ce-IS*1272* were IRs or “non-IRs”. IS*1272* included a larger structure with tandem duplications of the left (IR_L_) side sequence; *tnp* included minor cases of a long fusion form and truncated form. One ce-IS*1272* was associated with the segments responsible for immune evasion and drug resistance. Regarding virulence, GN1 expressed cytolytic peptides (phenol-soluble modulin α and δ-hemolysin) and PVL more strongly than some other familial strains. These results suggest that IS*1272* transposes through an IR-replacing mechanism, with an irreversible process unlike that of “canonical” transpositions, resulting in genomic variations, and that, among the familial strains, the patient strain has strong virulence potential based on community-associated virulence factors.

## Introduction

*Staphylococcus aureus* is a common human pathogen that colonizes the nasal mucosa and skin and causes a wide spectrum of diseases, including skin and soft tissue infections (SSTIs) such as furuncles and cellulitis, systemic infections including bacteremia, sepsis, osteomyelitis, bacteremic pneumonia, and endocarditis, and exotoxin-related diseases such as toxic shock syndrome and food poisoning [1–5]. Most community-associated infections of *S*. *aureus* in the United States are those that affect skin and soft tissues [4]. *S*. *aureus* also poses a threat because many of its strains are drug-resistant, most notably methicillin-resistant *S*. *aureus* (MRSA) with staphylococcal cassette chromosome *mec* (SCC*mec*) [6], which emerged as healthcare-associated MRSA (HA-MRSA) in the early 1960s and as community-associated MRSA (CA-MRSA) in the late 1990s [7–10].

*S*. *aureus*, both methicillin-susceptible *S*. *aureus* (MSSA) and CA-MRSA, often produce Panton-Valentine leukocidin (PVL), which is associated with pyogenic skin infections (large abscesses) [11–13]. PVL is cytotoxic against human polymorphonuclear neutrophils (PMN) and monocytes [14,15] and is encoded by a phage [16]. PVL also acts as a spread factor [9,12,17], with PVL-positive *S*. *aureus* and CA-MRSA often causing infections among family members through skin-to-skin contact [9,17,18]. The strong expression of peptide cytolysins, such as phenol-soluble modulins (PSMs) and δ-hemolysin (Hld), is also associated with CA-MRSA [12,19–22] and CA-MSSA [20,22].

Bacterial chromosomes typically have mobile DNA [23]. *S*. *aureus* mainly achieves its dynamic evolution through the action of mobile genetic elements, including insertion sequences (ISs), transposons (Tns), plasmids, phages, and *S*. *aureus* pathogenicity islands [6,23–27]. ISs are generally phenotypically cryptic and only carry the transposase gene (*tnp*), which is necessary for its intracellular transposition [23,28]. However, ISs occasionally exist as multiple copies in a cell [23,28,29]; they often act as mutators and play a role in large chromosomal rearrangements [23,29], the regulation of gene expression [23,26], homologous recombination [23,29], and gene deletion/disruption [23,29,30]. Historically, “canonical” ISs or Tns have been characterized by their terminal inverted repeats (IRs), which may form a stem-loop structure, and flanking target site duplications (TSDs) at both ends, which are generated upon transposition [23,28].

There are more than 4,500 different ISs (Isfinder, https://www-is.biotoul.fr/index.php). ISs have been divided into two major types based on transposases, specifically DDE and HUH enzymes, which catalyze the breaking and re-joining of DNA during insertion/transposition [23,28]. The common feature of DDE-type ISs (and Tns) is possessing terminal IRs, which serve as the binding site of DDE transposase [23,28]. DDE-type ISs (and Tns) include subtypes that utilize different intermediate formation mechanisms [28]. For example, IS*6* and Tn*3* have flanking direct repeats (TSDs) originating from the short target sites of 8 bp [31] and 5 bp [32], respectively, which were created through cointegrate formation (or target-primed transposon replication) [28]. Furthermore, IS*630* targets a 5’-NTAN sequence and achieves insertion in a cut-and-paste manner [28]. Previously identified target sequences are all non-IR sequences, and no IR targets have yet been identified.

IS*1272* was initially described in *S*. *haemolyticus*; it is 1,934 bp in size with 16-bp terminal IRs (sequence identity, 15 of 16 bp), lacks flanking TSDs, and contains two open reading frames (*orf*s): *orf1* and *orf2* [33]. Nosocomial isolates of *S*. *haemolyticus* are rich in IS*1272* copies, suggesting the potential role of IS*1272* in bacterial virulence [34]; however, whole-genomic and virulence information are currently lacking to confirm this. The initial IS*1272* study [33] also showed that *S*. *aureus* contains IS*1272*, but these sequences are mostly incomplete fragments of IS*1272*, suggesting that IS*1272* originally resided in *S*. *haemolyticus* and that MRSA has more IS*1272*-hybridizing fragments than MSSA. The MRSA type IV SCC*mec* (SCC*mec*IV) possesses the *mecA*-Δ*mecR1*-ΔIS*1272* region [6]. In addition, the highly successful HA-MRSA, MRSA252 [35], carries nine copies of IS*1272* on its genome (GenBank accession number NC_002952). However, IS*1272* may not be common in MRSA; for example, USA300 (GenBank accession number PC000255; strain FPR3757), a successful PVL-positive CA-MRSA associated with large outbreaks, including serious invasive infections, in the United States in 2007 [7,36], has no copies of IS*1272* in its genome, whereas SCC*mec*IVa in USA300 carries a truncated IS*1272*, as described above. The PVL phages, including that of USA300 [36], generally do not have any copies of IS*1272* in their genomes (GenBank accession numbers PC000255).

During the course of a study on the PVL S/F genes and predicted PVL S/F amino acid sequences [37,38], we determined the sequences of not only the entire PVL gene but also of its upstream and downstream regions in clinical *S*. *aureus* isolates by PCR and sequencing. We detected an IS*1272* transposition onto the PVL-converting prophage and proposed the idea of a unique “stem-loop replacing” mode of transposition, in which a target “stem-loop” structure is replaced by that of IS*1272* [39] (GenBank accession numbers AB256036-39). In the present study, we elucidated the sequences of the complete circular genomes using the patient strain (GN1) and parent strain (GN3), clinically isolated from a family infection case caused by a single clone of PVL-positive ST50 community-associated *S*. *aureus* (GN), and we performed a comprehensive comparison between the GN1 and GN3 genomes. This evaluation focusd on the status of multiple IS*1272* copies in the *S*. *aureus* genomes, including newly-inserted IS*1272* (ni-IS*1272*); we unambiguously revealed that the targets of transposition for IS*1272* were IR sequences (with different sequences and sizes), in contrast with the targets of previously describe ISs (“canonical” or not). For commonly-existing IS*1272* (ce-IS*1272*) in GN1 and GN3, we searched the target site sequences using a database of previously reported genome sequences. In light of our results, we discuss, particularly from the viewpoint of basic science, potential mechanisms that could be responsible for IS*1272* transposition, given that IS*1272* generates a deletion of its target “stem-loop” structure.

In previous molecular epidemiology studies of clinical *S*. *aureus* infections, one clonal infection of *S*. *aureus* lineage was verified, for example, by pulsed-field gel electrophoresis (PFGE) analysis of the genomes [17,40]; however, the whole genomes of *S*. *aureus* in this type of familial infection case have not been elucidated sufficiently, particularly in terms of the IS transposition and virulence potential. Therefore, in the present study, we analyzed variations among the GN genomes as well as virulence potential differences among GN familial strains, particularly for community-associated factors, peptide cytolysins and PVL.

## Materials and methods

### Ethics statement

The Ethics Review Board of Niigata University School of Medicine, Niigata, Japan (Ethics Review Board No. 748) specifically approved this study. Written informed consent was obtained from patients, where necessary.

### A patient, familial infections, and bacterial strains

**Fig 1:**
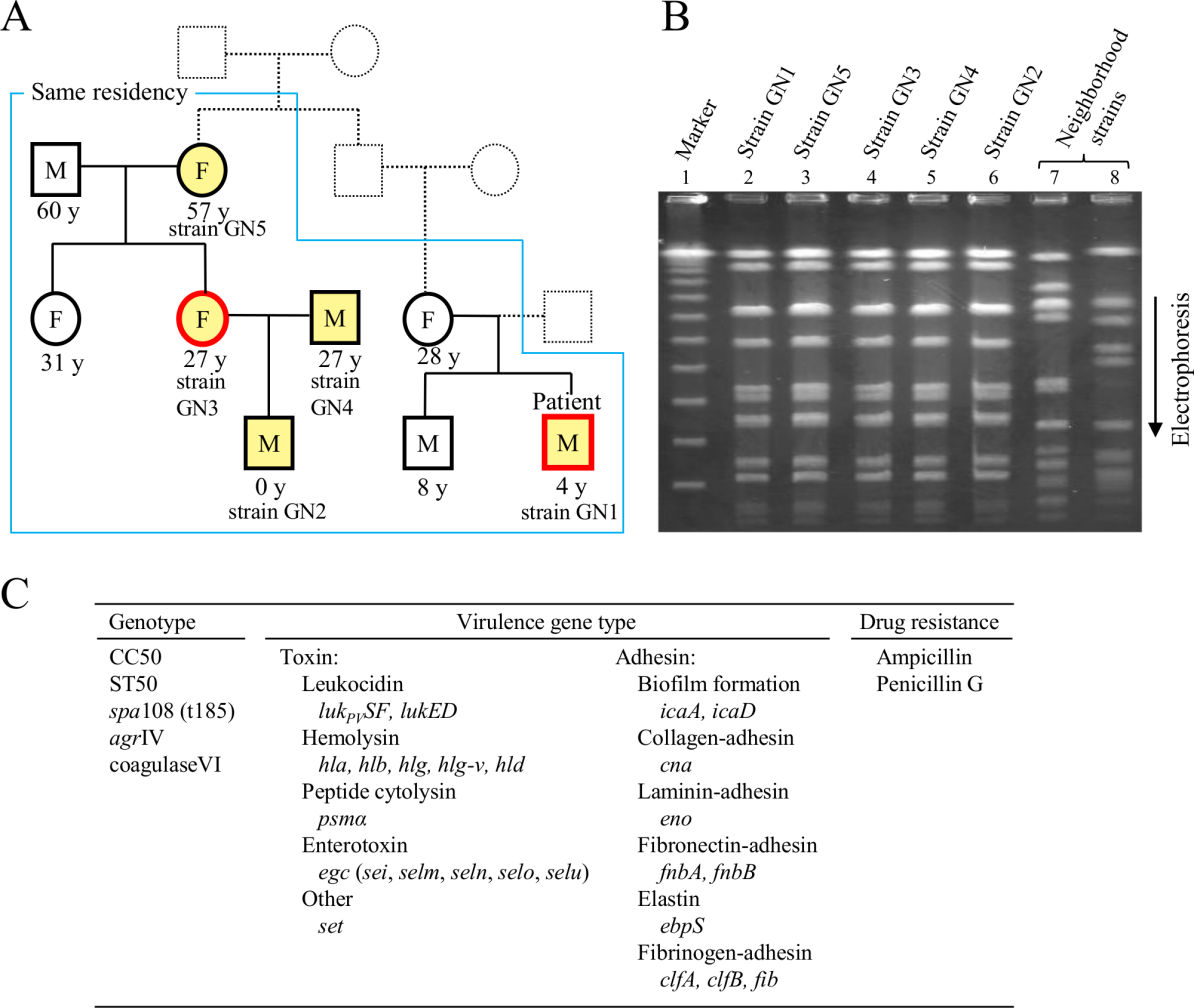
Intrafamilial transmission and molecular characteristics of Panton-Valentine leukocidin (PVL)-positive *Staphylococcus aureus*. (A) Nine family members (from three families) were living together with the 4-year-old boy (a patient who had skin abscesses) within the same house. Five members who were infected with PVL-positive *S*. *aureus* are marked with yellow. Four members, except the 4-year-old patient, did not develop any symptoms. Red square and circle indicate that the gonomes of PVL-positive *S*. *aureus* were sequenced. PVL-positive *S*. *aureus* srains: GN1, patient strain; GN2, infant strain; GN3, 27-year-old parent (female) strain; GN4, 27-year-old parent (male) strain; GN5, strain from the oldest member among infected members. (B) Familial strains (GN1 to GN5) were analyzed by pulsed-field gel electrophoresis, suggesting a clonal infection from one PVL-positive *S*. *aureus* source. Neighborhood strains were from healthy persons from neighboring families unrelated to the patient's families. (C) The genotypes, vurulence genes analyzed by PCR, and drug resistance of familial strains GN1 to GN5 are summarized, indicating the common features among strains GN1 to GN5.

A 4-year-old boy was brought to a hospital (Kido Hospital, Niigata) on July 23, 2005. Large skin abscesses (furuncles) were observed in his gluteal region. PVL-positive CA-MSSA was isolated from pus; this strain was designated GN1. The epidemiological definition of CA-*S*. *aureus* was based on the Centers for Disease Control and Prevention (CDC) criteria for CA-MRSA and HA-MRSA [7]. A 10% zinc oxide ointment sheet was placed on the furuncle region, and the patient was orally administered clarithromycin at 100 mg/day. The patient had frequently developed SSTIs including skin abscesses between 2003 and 2004. Nasal swabs were obtained in 2005 from eight of the patient’s family members (from three families) who were living together with the boy within the same house to examine familial infections (Fig 1A). PVL-positive MSSA was isolated from four out of the eight family members; therefore, a total of five out of the nine members (including the patient) were positive for PVL-positive MSSA, and their ages ranged from 0–60 years with a mean age of 26.9 years. Except for the 4-year-old boy (patient), the infected individuals did not show any symptoms; the PVL-positive MSSA isolated from these individuals were designated GN2 to GN5, as shown in Fig 1A. Two colonies each of GN1 to GN5, developed on initial bacterial isolation agar media, were characterized for *S*. *aureus* genotypes and drug susceptibilities.

Twenty-nine MSSA and two MRSA strains were also isolated from the nasal swabs of 78 healthy neighbors (including children) from 21 families (unrelated to the patient family); they were all PVL-negative.

### Genotyping and virulence gene analysis

Multi-locus sequence typing (MLST) was performed as described previously [21,41]; the ST type was obtained from the MLST website (http://www.mlst.net/). The *spa* (protein A gene) type was analyzed by PCR and elucidated using the public *spa* type databases eGenomics (http://tools.egenomics.com/) and Ridom SpaServer (http://spaserver.ridom.de/). The accessory gene regulator (*agr*) allele group was assessed by performing PCR with previously reported primers [39,40]. Coagulase typing was conducted using a staphylococcal coagulase antiserum kit (Denka Seiken, Tokyo, Japan), according to the manufacturer's instructions. An analysis of virulence genes was performed based on PCR results [21,29,41]. This analysis included 48 virulence genes: 3 leukocidin genes (*luk*_*PV*_*SF*, *lukE-lukD*, and *lukM*), 5 hemolysin genes (*hla*, *hlb*, *hlg*, *hlg-v*, and *hld*), the peptide cytolysin (PSMα) gene (*psmα*), 19 staphylococcal superantigen (SAg) genes, named enterotoxin (SE) or enterotoxin-like (SEl) genes (*tst*, *sea*-*e*, *seg*-*j*, *selk*-*r*, and *selu*), staphylococcal exotoxin (*set*) genes, a staphylococcal superantigen-like gene cluster (*ssl*), 3 exfoliative toxin genes (*eta*/*b* and *etd*), the epidermal cell differentiation inhibitor gene (*edin*), and 14 adhesin genes (*icaA*/*D*, *eno*, *fib*, *fnbA*/*B*, *ebpS*, *clfA*/*B*, *sdrC*-*E*, *cna*, and *bbp*).

### Susceptibility testing

Susceptibility testing of bacterial strains was performed using the agar dilution method with Muller-Hinton agar according to previously described procedures [42]. Thirty-nine antimicrobial agents were tested, including 15 β-lactams, 4 aminoglycosides, 3 tetracyclines, 3 macrolides, 3 fluoroquinolones, and 2 glycopeptides, as well as lenezolid, clindamycin, trimethoprim, sulfamethoxazole, chloramphenicol, fosfomycin, mupirocin, rifampicin, and fisidic acid. Breakpoints for drug resistance were those described by the Clinical and Laboratory Standards Institute (CLSI) [42].

### PFGE analysis

Bacterial DNA was digested with *Sma*I, and digested DNA was applied to PFGE (1.2% agarose), as described previously [17,21,29,40]. A lambda ladder (Bio-Rad Laboratories, Tokyo, Japan) was used as the molecular size standard (marker).

### Genome analysis

**Fig 2:**
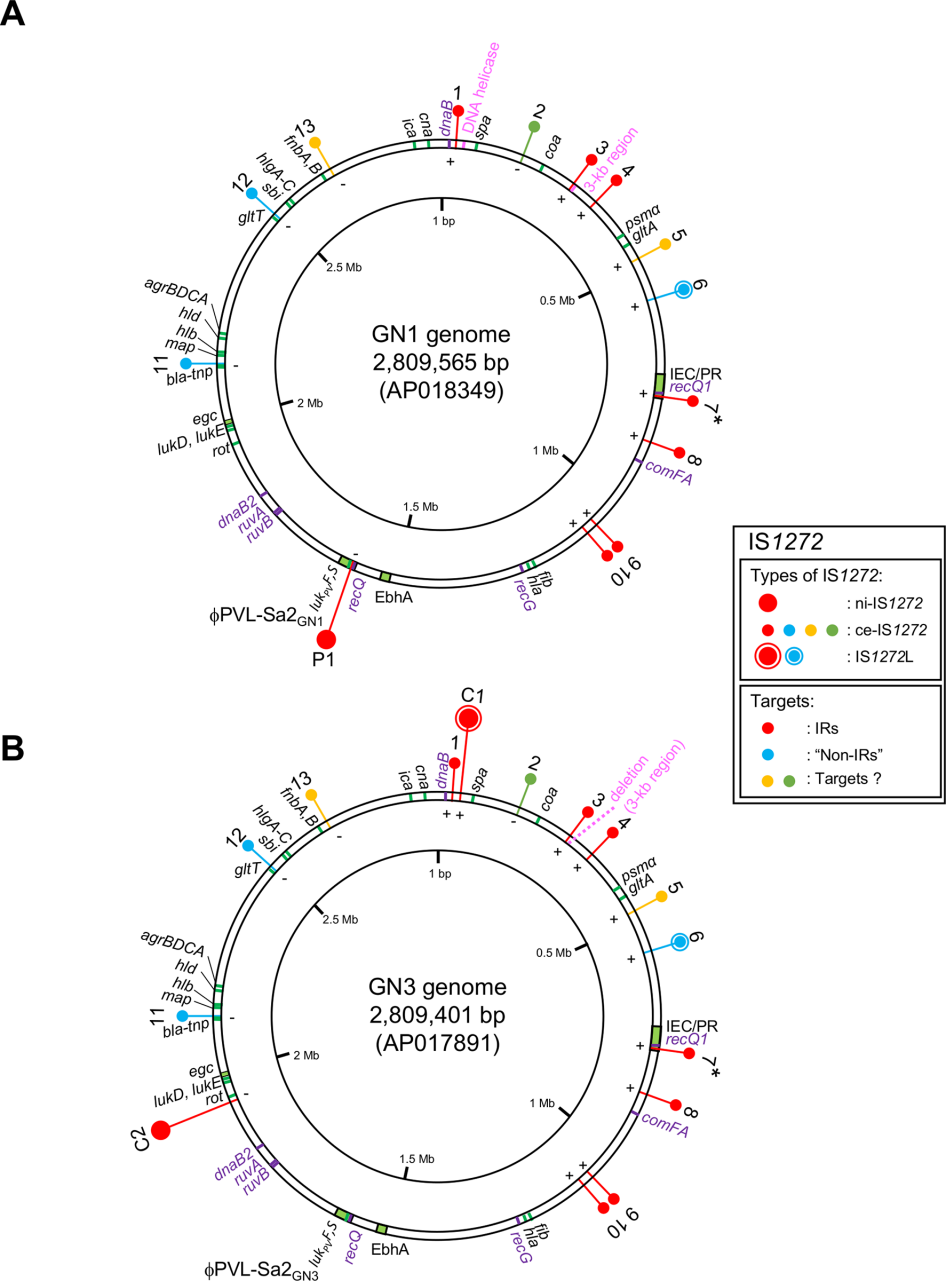
Circular genome maps of familial strains GN1 and GN3. Genomes: A, GN1; B, GN3. Genome information includes *S*. *aureus*-typing targets, phages, mobile genetic elements, including IS*1272*, deletion, virulence, and drug resistance. Genes (products) described on the genome map are: *spa*, protein A (IgG-binding protein); *coa*, coagulase; *psma*, phenol-soluble modulin *α*(PSM*α*, cytolytic peptide); *gltA*, glutamate synthase; *fib*, fibrinogen adhesin; *hla*, *α*-hemolysin (Hla); *ebhA*, extracellular matrix-binding protein/very large surface-anchored protein/giant protein (Ebh); *rot*, repressor of toxins; *lukE-lukD*, bi-component leukocidin; *egc*, enterotoxin gene cluster carrying *sei*, *selm*, *seln*, *selo*, and *selu*; *map*, map protein; *hlb*, β-hemolysin (Hlb); *hld*, δ-hemolysin (Hld, cytolytic peptide); *agr*, accessory gene regulator; *gltT*, proton/sodium-glutamate symport protein, *sbi*, second binding protein of immunoglobulin, *hlg*, γ-hemolysin (Hlg); *fnb*, fibronectin-binding protein; *ica*, intercellular adhesion protein A (biofilm formation); *cna*, collagen adhesin. The DNA helicase gene and eight other helicase genes, *dnaB*, *recQ1*, *comFA*, *recG*, *recQ*, *ruvB*, *rubA*, and *dnaB2*, were also mapped on the genomes; they are shown in purple. GN1 and GN3 had PVL-converting φSa2 (φPVL-Sa2_GN1_ and φPVL-Sa2_GN3_, respectively), carrying the PVL genes (*luk*_*PV*_*SF*). Unique genetic structures were carried by φSa3 remnant, the immune evasion cluster (IEC), composed of three immune evasion genes, *sak* (staphylokinase, SAK), *chp* (chemotaxis inhibitory protein of *S*. *aureus*, CHIPS), and *scn* (staphylococcal complement inhibitor, SCIN), being present on the GN genome as IEC/PR. Penicillin resistance was encoded by a chromosomal *bla-tnp* structure, carrying an array *of blaI-blaR1-blaZ and tnpC-tnpB-tnpA*. The GN1 and GN3 genomes carried 14 and 15 copies of IS*1272*, respectively. Of those, 13 copies were commonly-existing IS*1272* (ce-IS*1272*), and named 1 to 13; moreover, GN1 had one copy of newly-inserted IS*1272* (ni-IS*1272*), which was named P1, while GN2 had two copies of ni-IS*1272*, which were named C1 and C2, as shown in A and B, respectively. ce-IS*1272* copy 6 and ni-IS*1272* copy C1 formed a larger structure (designated as IS*1272*L). ce-IS*1272* copy 2, labeled in green, had the transposase gene (*tnp*) encoding for a long fusion form of transposase; ce-IS*1272* copy 7, marked with an asterisk, had *tnp* encoding for a truncated form of transposase. The direction of the IS*1272* insertion is shown by + or -. The targets of IS*1272* transposition are indicated by color: red, IRs; blue, “non-IRs”; yellow and green, unknown (target sequences are too big in size). The 3-kb region, located close to IS*1272* copy 3, in GN1 (A) was deleted in GN3 (B).

The PVL prophage genomes and bacterial genomes of GN1 and GN3 (patient and mother strains, respectively, Fig 1A) were analyzed. The entire genome sequences of the PVL prophages contained by GN1 and GN3, named φPVL-Sa2_GN1_ and φPVL-Sa2_GN3_, respectively, were determined by PCR and sequencing; the genome sizes were 48,010 bp and 46,089 bp, respectively, and the GenBank accession numbers are LC086374 and LC086375, respectively. The bacterial genome sequences of GN1 and GN3 were analyzed by a long-read single-molecule real-time (SMRT) sequencing platform with P5/C3 chemistry using sequencing technology and the PacBio RS II system (Pacific Biosciences, Menlo Park, CA, USA) with the assembler software SMRT Analysis v2.3.0/hierarchical genome-assembly process (HGAP) pipeline [43]. Genome coverage (sequencing depth) was 563-fold and 443-fold of each genome size for GN1 and GN3, respectively. Completion of the genome contig to construct the full circular genome sequence was performed by PCR and sequencing. For the GN1 and GN3 bacterial genomes, the sizes were 2,809,565 bp and 2,809,401 bp, respectively (Fig 2), and the GenBank accession numbers are AP018349 and AP017891, respectively.

### PCR analysis of IS*1272* insertions in the PVL prophage region of familial strains

Nine PCR primer sets (A to C and 1 to 6) were used to investigate the presence of an IS*1272* insertion at a region located downstream of the PVL S/F genes (*luk*_*PV*_*SF*) on the PVL prophage DNA; primer sequences are summarized in S1 Fig [37,44]. PCR primers, PVL-1 and NPVL-2, were based on reference [44]. Other PCR primers were initially designed based on the DNA sequence of a PVL prophage lacking an IS*1272* insertion that was carried by the ST30 CA-MRSA strain NN1 [37]; later primers were designed based on the sequences of φPVL-Sa2_GN1_ and φPVL-Sa2_GN3_. The primers for IS*1272* were designed based on the φPVL-Sa2_GN1_ sequence, which has an IS*1272* insertion. Of the three primer sets (A to C), primer set B detects the terminal IRs of the target sequence of IS*1272*; thus, in the present study, GN1, GN2, and GN5 (in which the target was replaced by IS*1272*) each produced negative results in PCR assays using primer set B. Of the othe six PCR primer sets (1 to 6), primer set 1 detects the PVL S gene and also its upstream region (thus, in the present study, GN1 to GN5 each gave PCR products of the same size, indicating no IS*1272* insertion); primer sets 2–5 each detect an IS*1272* inserted downstream of the PVL S/F genes (thus, in the present study, GN1, GN2, and GN5 each produced positive results); and primer set 6 detects the 3'-end region of the PVL F gene and a region located downstream of the PVL F gene (thus, in the present study, GN1 to GN5 all yielded PCR products, but GN1, GN2, and GN5 each produced ca. 2-kb bigger PCR products, due to the presence of an IS*1272* insertion).

### mRNA expression assay

*S*. *aureus* strains were cultured on 5% sheep blood agar (Becton Dickinson, Tokyo, Japan) for 8 h at 37°C. The mRNA expression levels of the *psmα*, δ-hemolysin (*hld*), PVL (*luk*_*PV*_*SF*), and 16S rRNA genes were then examined using an RT-PCR assay [20–22]. The *psmα hld*, and *luk*_*PV*_*SF* expression levels were then normalized to 16S rRNA expression levels. ST5/SCC*mec*II HA-MRSA strains (Mu50 and N315) were used as low *psmα hld* expression control strains, and the ST8/SCC*mec*IVa PVL^+^ CA-MRSA type strain USA300-0114, ST30/SCC*mec*IVc PVL^+^ CA-MRSA strain RS08, and ST121/*agr*4 CA-MSSA strain KT1 were used as stronger *psmα*/*hld* expression control strains [20–22]. Experiments were repeated six times for each strain.

### PVL assay

*S*. *aureus* strains were cultured in brain heart infusion (BHI) broth (Becton Dickinson, Sparks, MD, USA) with or without of 5% fetal bovine serum (Gibco, Carlsbad, CA, USA) for 18 h at 37°C; resultant cultures were adjusted to an optical density of 600 nm (OD_600_) of approximately 0.7 (at 10-fold dilutions). Serial doubling dilutions of the culture supernatants were made, and the amounts of PVL in the supernatants of bacterial cultures were examined using a PVL- RPLA kit (Denka Seiken, Niigata, Japan), according to the instructions of the manufacturer. Experiments were repeated four times for each strain.

### Phylogenetic analysis

Multiple alignments were performed up to 1,000 times using default settings with ClustalW software (version 2.1), and a phylogenetic tree analysis was performed using TreeViewX software (version 0.5.0) (http://taxonomy.zoology.gla.ac.uk/rod/treeview.html).

### Analysis of the target sequences of ce-IS*1272* transposition

A database composed of previously reported *S*. *aureus* genomes was searched for target sequences for ce-IS*1272* transposition. The analysis of homology between the *S*. *aureus* genome sequences containing the target sequences of ce-IS*1272* transposition and the GN1/GN3 genome sequences carrying ce-IS*1272* was performed using the software BLAST (http://blast.ddbj.nig.ac.jp/top-e.html).

### PCR analysis of a possible extrachromosomal circular DNA molecule of IS*1272*

In order to investigate a possible extrachromosomal circular DNA molecule of IS*1272*, we designed PCR primers, IS1272-0F (5'-AAGACCGAGGCTGAGACG) and IS1272-0R (5'-GGAAAATAGCAGCTCGACG), based on the IS*1272* sequences in GN1 and GN3.

### Statistical analysis

Data were evaluated by a Student’s *t*-test, a Fisher’s exact test, or an analysis of variance with repeated measurements for the mRNA expression assay. The level of significance was defined as a *P* value of <0.05.

## Results

### Familial infection from PVL-positive MSSA

Among the nine members from three families who were living together within the same house, five were positive for PVL-positive CA-MSSA, and the strains isolated from these individuals were named strains GN1 to GN5 (Fig 1A). A PFGE analysis revealed that the five PVL-positive CA-MSSA strains GN1 to GN5 were the same (Fig 1B), indicating the intrafamilial spread of the single PVL-positive CA-MSSA clone (GN). The PVL-positive CA-MSSA GN (strains GN1 to GN5) belonged to ST50 (CC50), exhibited *spa*108 (t185), *agr*4, and coagulase VI, carried toxin genes such as *luk*_*PV*_*SF*, *hld*, *psmα* and *egc* (an entrotoxin gene cluster carrying *sei*, *selm*, *seln*, *selo*, and *selu*, but lacking *seg*), carried 12 adhesin genes including *cna* (encoding for collagen adhesin), and was only resistant to ampicillin/penicillin G (Fig 1C). The above characteristics of GN strains (GN1 to GN5) were confirmed for two initially-isolated colonies of each strain.

Among the five family members infected with GN, only a 4-year-old boy, infected with strain GN1, developed furuncles, whereas the four other members, a <1-year-old infant, 27-year-old female and male parents, and the oldest (57-year-old) infected member (infected with strains GN2, GN3, GN4, and GN5, respectively), did not develop any symptoms.

PVL-positive MSSA was not isolated from the healthy individuals of neighboring families; MSSA (or MRSA) strains isolated from neighboring family members were divergent, as shown in Fig 1B.

### The complete circular genome structures of patient strain GN1 and parent strain GN3

The GN1 and GN3 genomes were estimated to be 2,809,565 bp and 2,809,401 bp, respectively. Based on the GN1 and GN3 complete circular genome sequences, the GN1 and GN3 circular genome maps were constructed, as shown in Fig 2A and 2B, respectively, with a focus on IS*1272*, some virulence genes, some regulatory genes or regulons, genes used for genotyping (*spa*, *agr*, and *coa*), resistance genes, and phages.

Regarding phages, the GN1 and GN3 genomes each carried PVL-converting φSa2. These phages, φPVL-Sa2_GN1_ and φPVL-Sa2_GN3_, were 48,010 bp and 46,089 bp in size, respectively, and showed 85% and 98% homology with the PVL-converting φSa2 of JCSC7401/ST80 MRSA (S2 Fig). The overall homology between φPVL-Sa2_GN1_ and φPVL-Sa2_GN3_ was 95.9%. GN1 and GN3 both lacked φSa3, φSa5, φSa6, and φSa7.

Regarding ISs, 14 and 15 copies of IS*1272* were distributed along the GN1 and GN3 genomes, respectively. In addition to IS*1272*, the GN1 and GN3 genomes carried several other ISs: eight copies of ISSep3, designated as ISSep3 (GN1) or (GN3), and two copies of ISSep2, designated as ISSep2 (GN1) or (GN3), suggesting that IS*1272* was the most prevalent IS in the GN1 and GN3 genomes. GN1 and GN3 did not have any copies of IS*256*, which exhists as a multi-copy system in epidemic MRSA [29].

Regarding virulence genes, the immune evasion cluster (IEC), which is generally present on the left-end side of φSa3 [21,27,29,45], was found in the GN1 and GN3 genomes. IEC (GN1) and IEC (GN3) each carried three immune evasion genes: *sak* for staphylokinase (SAK), *chp* for the chemotaxis inhibitory protein of *S*. *aureus* (CHIPS), and *scn* for staphylococcal complement inhibitor (SCIN), as well as the φSa3 remnant. Therefore, the IEC in the GN1 and GN3 genomes was designated as IEC/PR. The location of IEC/PR was not *hlb*, which provides the insertion site (*att*) for φSa3; IEC/PR was located distant from *hlb*. Thus, the *hlb* in the GN1 and GN3 genomes was intact (Figs 1C, 2A and 2B).

Regarding resistance, three penicillin resistance-related genes, *blaZ*, *blaR1*, and *blaI*, which are carried by a penicillinase plasmid in *S*. *aureus* [46], were present in the GN1 and GN3 genomes.

### Status of multiple IS*1272* copies on the GN1 and GN3 genomes

**Fig 3:**
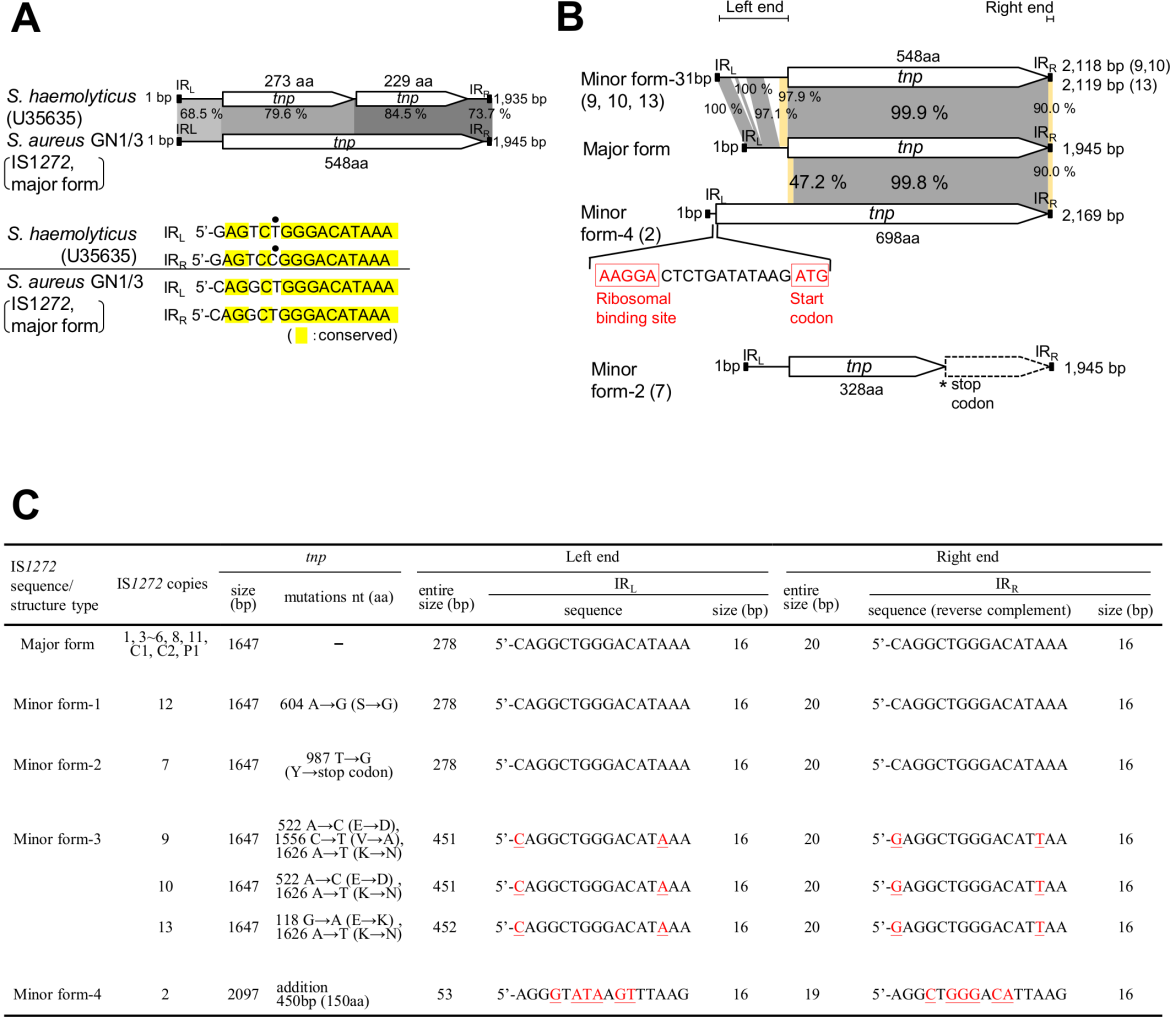
**Comparison of IS*1272* from *S*. *haemolyticus* and *S*. *aureus* GN (A), and comparison between IS*1272* copies on the GN genomes (B and C).** (A) The IS*1272* of *S*. *haemolyticus* has two transposase (Tnp) genes (ORFs, *tnp*) and heterogenous IRs (sequence identity, 15 of 16 bp); divergent nucleotides are shown by a dot [33]. In contrast, a major form of IS*1272* in GN1 and GN3 had only one *tnp* and homogeneous IRs (sequence identity, 16 of 16 bp). (B) The structures of IS*1272*, major form and minor forms (2, 3, and 4), were compared. Homologous regions are shaded in each comparison. IS*1272*/minor form-2 (copy 7) had a premature stop codon in *tnp*, thus its product was predicted to be truncated Tnp. IS*1272*/minor form-4 (copy 2) had *tnp* of a larger size, which started at an ATG codon located upstream of *tnp*/major form; the ribosome binding sequence (AAGGA), which can potentially pair with the complementary sequence at the 3'-end of 16S rRNA [47], is shown in red. (C) The genetic statuses of 16 IS*1272* copies on the GN1/GN3 genomes are summarized. IS*1272*/major form had 1,647-bp (548-aa) *tnp* and 16-bp homogeneous IRs (sequence identity, 16 of 16 bp). IS*1272*/minor form-1 had a nonsynonymous substitution in *tnp*. IS*1272*/minor form-2 had a premature stop codon in *tnp*. IS*1272*/minor form-3 had 16-bp heterogeneous IRs (sequence identity, 14 of 16 bp) and *tnp* which was divergent in comparison with *tnp*/major form. IS*1272*/minor form-4 had 16-bp more-divergent IRs (sequence identity, 10 of 16 bp) and *tnp* with a 450-bp (150-aa) longer N-terminal side. nt, nucleotide.

The IS*1272* copies of GN1 and GN3, IS*1272* (GN1) and IS*1272* (GN3), only carried one transposase gene (*tnp*), unlike IS*1272* (*S*. *haemolyticus*), which has two *orf*s [33], as shown in Fig 3A [33,47]. The *orf1* sequence of *S*. *haemolyticus* IS*1272* contains a premature stop codon due to a frame shift caused by a one-base deletion, relative to the transposase gene sequences of IS*1272* (major form) of GN1 and GN3. Regarding the 16-bp terminal IR sequences, IS*1272* (*S*. *haemolyticus*) and IS*1272*-major form (GN1, GN3) were divergent by two nucleotides. The IR_L_ and IR_R_ of IS*1272* (*S*. *haemolyticus*) are heterogenous with a sequence identity of 15/16 [33], while the IR_L_ and IR_R_ of IS*1272*-major form (GN1, GN3) were the same (sequence identity, 16/16).

The 14 copies of IS*1272* (GN1) and 15 copies of IS*1272* (GN3) were not uniform. Based on the sequence divergence in the transposase gene and terminal IRs, the IS*1272* forms were classified into a major form and four minor forms (Fig 3B and 3C). The major form contained copies 1, 3–6, 8, 11, C1, C2, and P1 (Fig 2A and 2B); these copies encoded for 548-amino acid (aa) transposase, had terminal IRs of the same sequences (sequence identity of IR_L_ and IR_R_, 16/16), and showed an over all sequence identity of 100% (Fig 3B and 3C). Minor form-1 (copy 12) (Fig 2A and 2B) had a point mutation (nonsynonymous substitution, which caused an amino acid change) in the transposase gene (Fig 3C); minor form-2 (copy 7) (Fig 2A and 2B) had a premature stop codon in the transposase gene, resulting in a truncated (328-aa) transposase (Fig 3B and 3C); minor form-3 (copies 9, 10, and 13) (Fig 2A and 2B) had two or three nonsynonymous substitutions in the transposase gene and also heterogeneous terminal IRs (sequence identity of IR_L_ and IR_R_, 14/16) (Fig 3B and 3C); and minor form-4 (copy 2) (Fig 2A and 2B) used a new start codon for the transposase gene, resulting in a 150-aa larger (698-aa) transposase (Fig 3B), and also had heterogeneous terminal IRs (sequence identity of IR_L_ and IR_R_, 14/16) (Fig 3C).

**Fig 4:**
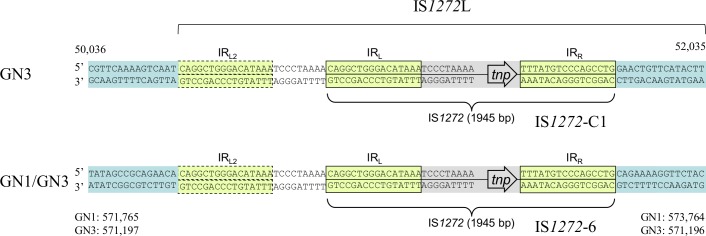
Structure of IS*1272* with tandem duplications of its left side sequence. ce-IS*1272* copy 6 in GN1 and GN3 and ni-IS*1272* copy C1 in GN3 formed a larger IS*1272* structure (designated as IS*1272*L) with the same sequence. IS*1272*L had tandem duplications of the 25-bp left side sequence including IR_L_; the size of IS*1272*L with IR_L2_ and IR_R_ is 1,970 bp.

IS*1272* copy C1, on the GN3 genome (Fig 2A), and IS*1272* copy 6, on both the GN1 and GN3 genomes (Fig 2A and 2B), formed a larger structure (IS*1272*L) with tandem duplications of the 25-bp left side sequence including IR_L_; IS*1272*L was 1,970 bp in size and had terminal IR sequence (IR_L2_) in addition to an ordinary major form of 1,945-bp IS*1272* with IR_L_ and IR_R_ (Fig 4). Unlike IS*256* [29,48], IS*1272* did not form an extrachromosomal circular DNA molecule.

### Targets and the mode of transposition for ni-IS*1272*

**Fig 5:**
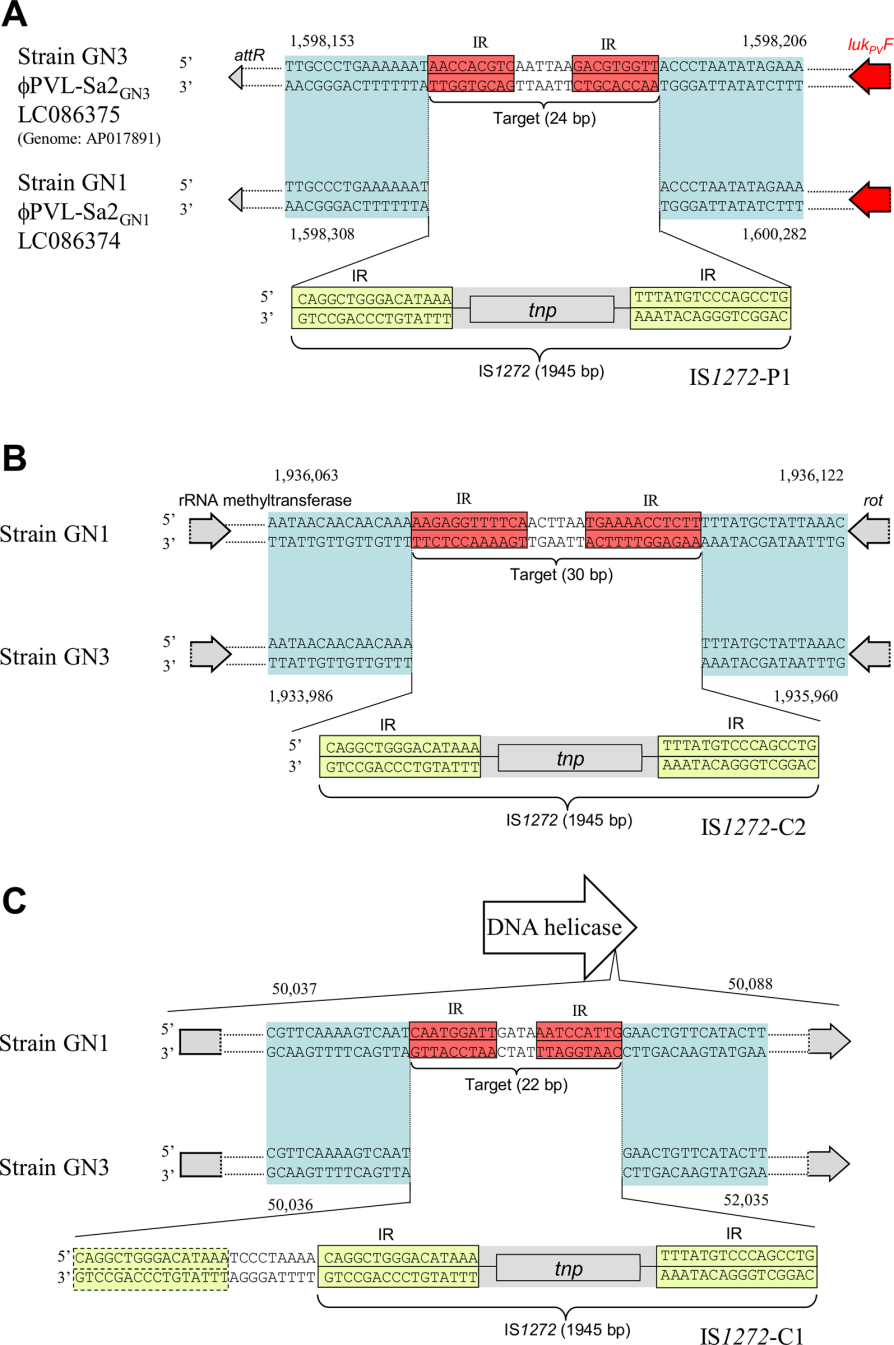
Evidence of inverted repeats (IR)-replacing transposition for IS*1272*. Comparison of GN1 and GN3 genome sequences at a position of newly-inserted IS*1272* (ni-IS*1272*) made it possible to definitely assign a set of target and IS*1272* insertion. Color: red, target inverted repeats (IRs); yellow, terminal IRs of IS*1272*; blue, the same DNA sequence region between GN1 and GN3. In A, IS*1272* transposition occurred on the PVL-prophage, targeting a 24-bp sequence with 9-bp IRs and yielding IS*1272*-P1 (on GN1). In B, IS*1272* transposition occurred in a region downstream of *rot* (and also downstream of the rRNA methyltransferase gene), targeting a 30-bp sequence with 12-bp IRs and yielding IS*1272*-C1 (on GN3). In C, IS*1272* transposition occurred within the DNA helicase gene, targeting a 22-bp sequence with 9-bp IRs and yielding the larger IS*1272* structure (IS*1272*L) of IS*1272*-C1 (on GN3); this IS*1272*L may have occurred by the tandem duplication of the 25-bp left side sequence upon transposition or by transposition of IS*1272*L. In all the three cases, the target IRs are replaced with IS*1272*.

A comparison of the GN1 and GN3 genome sequences revealed three cases of ni-IS*1272*; one case (copy P1) occurred in the GN1 genome and two cases (copies C1 and C2) in the GN3 genome (Fig 5). This new IS*1272* insertion on GN1 (copy P1 transposition) occurred at a 24-bp IR structure (with 9-bp terminal IRs and a 6-bp intervening region), which was located 66 bp downstream of the PVL F gene (*luk*_*pv*_*F*) in the prophage φPVL-Sa2 (GN3) region (Fig 5A, S2 Fig). One new IS*1272* insertion in GN3 (copy C2 transposition) occurred at a 30-bp IR structure (with 12-bp terminal IRs and a 6-bp intervening region), which was located 15 bp downstream of *rot* and also down stream of the rRNA methyltransferase gene (Fig 5B); the other new IS*1272* insertion in GN3 (copy C1 transposition) occurred at a 22-bp IR structure (with 9-bp terminal IRs and a 4-bp intervening region), which was located within the DNA helicase gene (at the 3’-end region) (Fig 5C). Thus, the targets of all three ni-IS*1272* transpositions were IR sequences, albeit with different sizes and different sequences, suggesting that the target is an IR structure (a potential stem-loop structure).

For all three cases, the insertion process may have included the complete separation of the IS*1272* IR structure from the flanking donor sequence, the insertion of IS*1272* into the target IR site, and the complete separation of the target IR structure from the flanking recipient sequence (“cut-paste-and-cut”), thereby resulting in an IR-replacing model of transposition (or potential stem-loop replacing model of transposition) (Fig 5). There were no TSDs for ni-IS*1272* in GN1 and GN3.

### Search for possible targets of ce-IS*1272*

**Fig 6:**
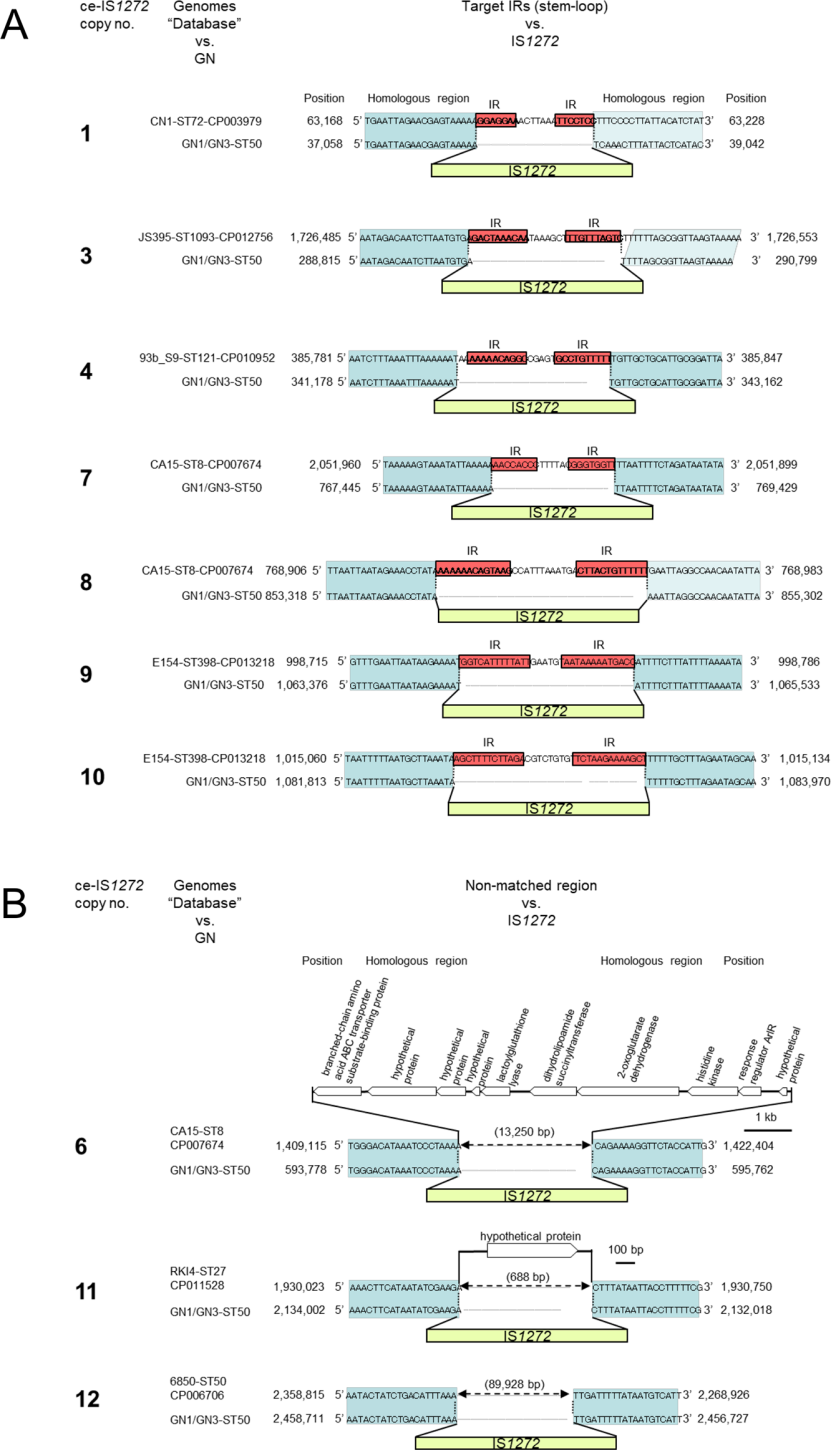
Analysis of previous transposition modes for current IS*1272* copies. For 13 copies of commonly-existing IS*1272* (ce-IS*1272*) on the GN1 and GN3 genomes, possible targets of transposition were searched in the database. Color: red, target inverted repeats (IRs); yellow, IS*1272* with terminal IRs; blue, the same DNA sequence region between the GN1/GN3 genome and the genome searched in the database. In A, the possible targets were IRs for seven copies of ce-IS*1272* (copies 1, 3, 4, 7–10). In B, the possible targets were “non-IRs” for three copies of ce-IS*1272* (copies 6, 11, 12). For the remaining three copies of ce-IS*1272* (copies 2, 5, 13), the possible targets were unknown (target sequences not specific or too big in size).

The GN1 and GN3 genomes each had 13 copies of ce-IS*1272* (Fig 2). To elucidate the targets and modes of the previous transpositions that produced the 13 current copies of ce-IS*1272*, a database of previously reported *S*. *aureus* genome sequences was searched for possible target sequences. Seven sets of possible target-containing “database” sequences and IS*1272*-containing GN1/GN3 sequences suggested the presence of a target IR structure and an IR-replacing mode of transposition, as shown in Fig 6A (for IS*1272* copies 1, 3, 4, 7–10).

**Fig 7:**
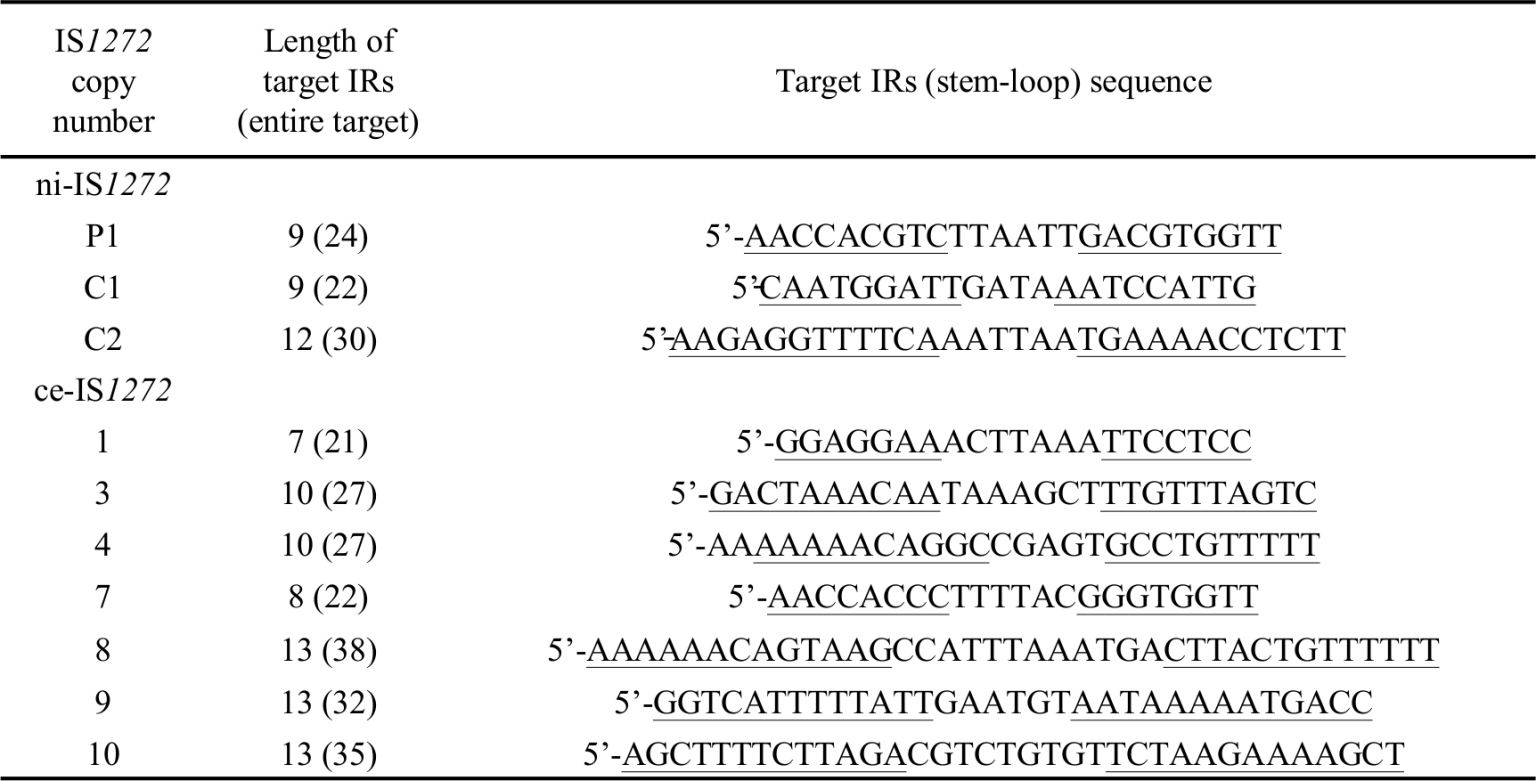
Target inverted repeat (IR) sequence analysis for IS*1272* on the GN genomes. For newly-inserted IS*1272* (ni-IS*1272*), target site sequences were analyzed by comparing the GN1 and GN3 genome sequences at each ni-IS*1272* insertion site. For commonly-existing IS*1272* (ce-IS*1272*), target site sequences were searched from the database. IRs in the target site sequences are underlined. The target site sequences exhibited no sequence homology to each other, suggesting the role of a stem-loop structure as a target of IS*1272* transposition.

Based on data from a total of 10 IS*1272* copies, ni-IS*1272* copies P1, C1, and C2 and ce*-*IS*1272* copies 1, 3, 4, and 7–10, the size of the target IR structure ranged between 21 and 38 bp, while that of the IRs ranged between 7 and 13 bp (Fig 7). In contrast, three ce-IS*1272* cases (6, 11, and 12) suggested that there were no IR structures as a target, as shown in Fig 6B; the mode of transposition is not clear for these cases. In the remaining three ce-IS*1272* cases (copies 2, 5, and 13), the target sequences were not specific or were too big in size in the present database searches.

### Database searches for the IS*1272* targets and transposition modes that support the IR-replacing mode of transposition

**Fig 8:**
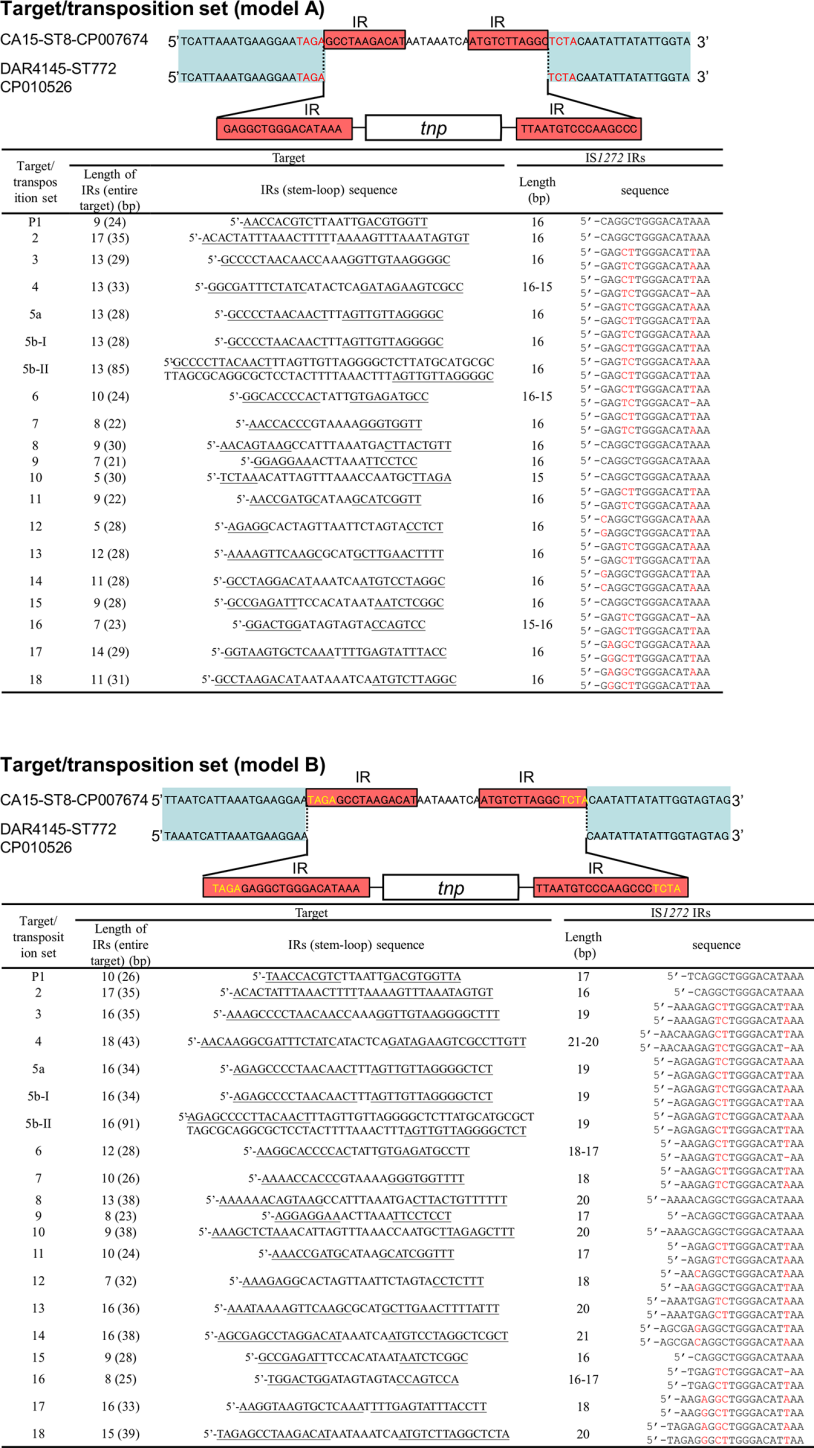
Target inverted repeat (IR) sequence analysis using target/IS*1272* sets obtained in database searches. The IR sequences from 19 target/IS*1272* sets were searched using the database and are summarized in figures. Those for IS*1272* copy P1 are from the GN1/GN3 genomes. Terminal IRs in the target sequences are underlined. IS*1272* IR sequences with red nucleotides represent heterogeneous IRs; red nucleotides are divergent in the left and right IRs. Model A shows the results when the size of IRs is 16 bp; model B shows the results when the size of IRs is 16 bp or more.

Database searches yielded 19 sets of IS*1272* target and transposition sequences, that support the IR-replacing mode of transposition, as shown in S3 Fig. Based on these results, the estimated target IR structure (target terminal IRs) and IS*1272* IRs are summarized in Fig 8. The length of IS*1272* IRs may be 16 bp (model A), yielding a target IR structure size that ranges between 21 and 85 bp and a target terminal IR size ranging between 5 and 17 bp. These results strongly indicate that the IS*1272* transposase recognizes targets as an IR structure (or a stem-loop structure), rather than as a specific sequence. Regarding the sequence of IS*1272* IRs, there was an IS*1272* copy P1 type (the major form shown in Fig 3C), however, further divergence was noted (Fig 8, model A).

Due to the two-IR (target and IS*1272*) nature, an alternate longer estimation of IS*1272* IR size may be possible (model B); in this model, the size of IS*1272* IRs ranged between 17 and 21 bp, while the size of target IR structures (and target terminal IRs) ranged between 23 and 91 bp (8 to 18 bp).

### Sizes of the IS*1272 tnp* genes

**Fig 9:**
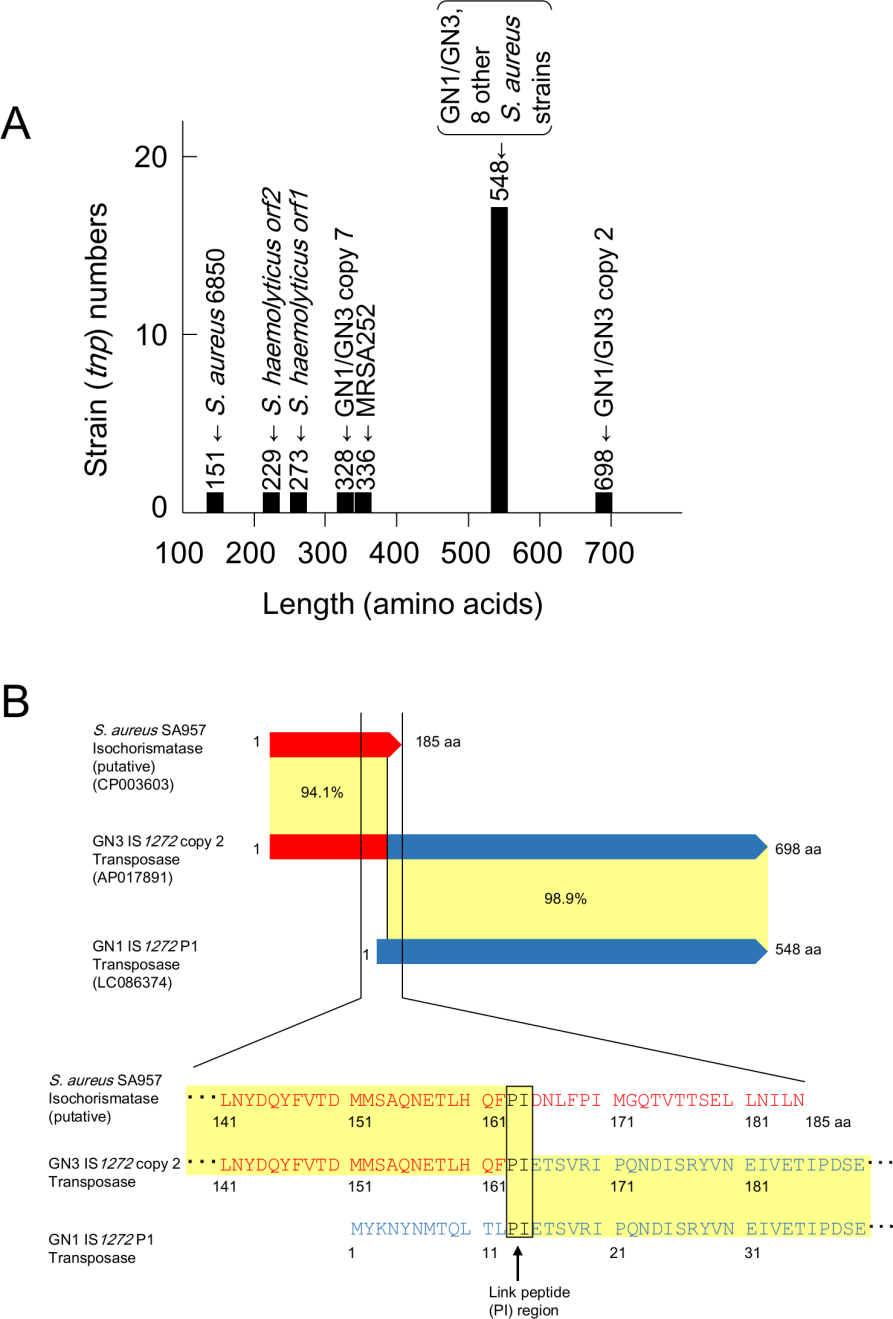
Variation in size of the IS*1272* transposase (Tnp) gene (*tnp*). (A) The sizes of the IS*1272 tnp* genes in GN1/GN3, other reported *S*. *aureus* (including MRSA), and *S*. *hemolyticus* are summarized in figures; other *S*. *aureus* included strains 6850, MRSA252, TCH60, JKD6159, SA268, T-ORC_001, SA957, and DAR4145 (they were from the database) and *S*. *haemolyticus* was from [33]. The majority of *tnp* genes encoded for a 548-aa product. However, for example, GN1/3 copy 7 *tnp* had a premature stop codon, resulting in a truncated product (328 aa); and *S*. *haemolyticus* IS*1272 tnp* had one base deletion, resulting in a frame shift mutation and two smaller ORFs (229 aa and 273 aa), due to one more deletion [31]. Moreover, GN1/3 copy 2 *tnp* encoded for a larger product (698 aa). (B) This figure shows that the *tnp* gene of GN1/3 IS*1272* copy 2 encodes for a fusion protein, constructed by isochorismatase (putative), shown in red, and GN1 IS*1272* copy P1 transposase, shown in blue. There was a link peptide region (PI), shown in black, between the isochorismatase (putative) domain and IS*1272* P1 transposase domain; the same link peptide region was also present in both isochorismatase (putative) and IS*1272* P1 transposase; the nucleotide sequence corresponding to the link peptide region was 5’-CCAATA.

The sizes of the IS*1272 tnp* genes analyzed in the present study are summarized in Fig 9A [33]. The majority of the *tnp* genes encoded for a 548-aa transposase. However, there were two cases of a premature stop codon, resulting in truncated transposases (*S*. *haemolyticus* IS*1272* and IS*1272*-copy 7) and one case of a larger sequence (IS*1272*-copy 2), as also shown in Fig 3A and 3B.

The transposase of IS*1272*-copy 2 was a fusion transposase, which was constructed by isochorismatase (putative) and IS*1272* transposase (Fig 9B). There was a link peptide region (PI) between the isochorismatase (putative) domain and IS*1272* transposase domain that was also shared by isochorismatase (putative) and IS*1272* transposase. The nucleotide sequence corresponding to the link peptide region (PI) was 5’-CCAATA in any case, providing a possible hot spot sequence for the genetic fusion event. The GN1 and GN3 genomes both lacked the isochorismatase (putative) gene, suggesting that the genetic fusion event occurred in other bacterial cells or that the isochorismatase (putative) gene was deleted in GN1 and GN3.

### Characteristic distribution of IS*1272* on the GN genomes

**Fig 10:**
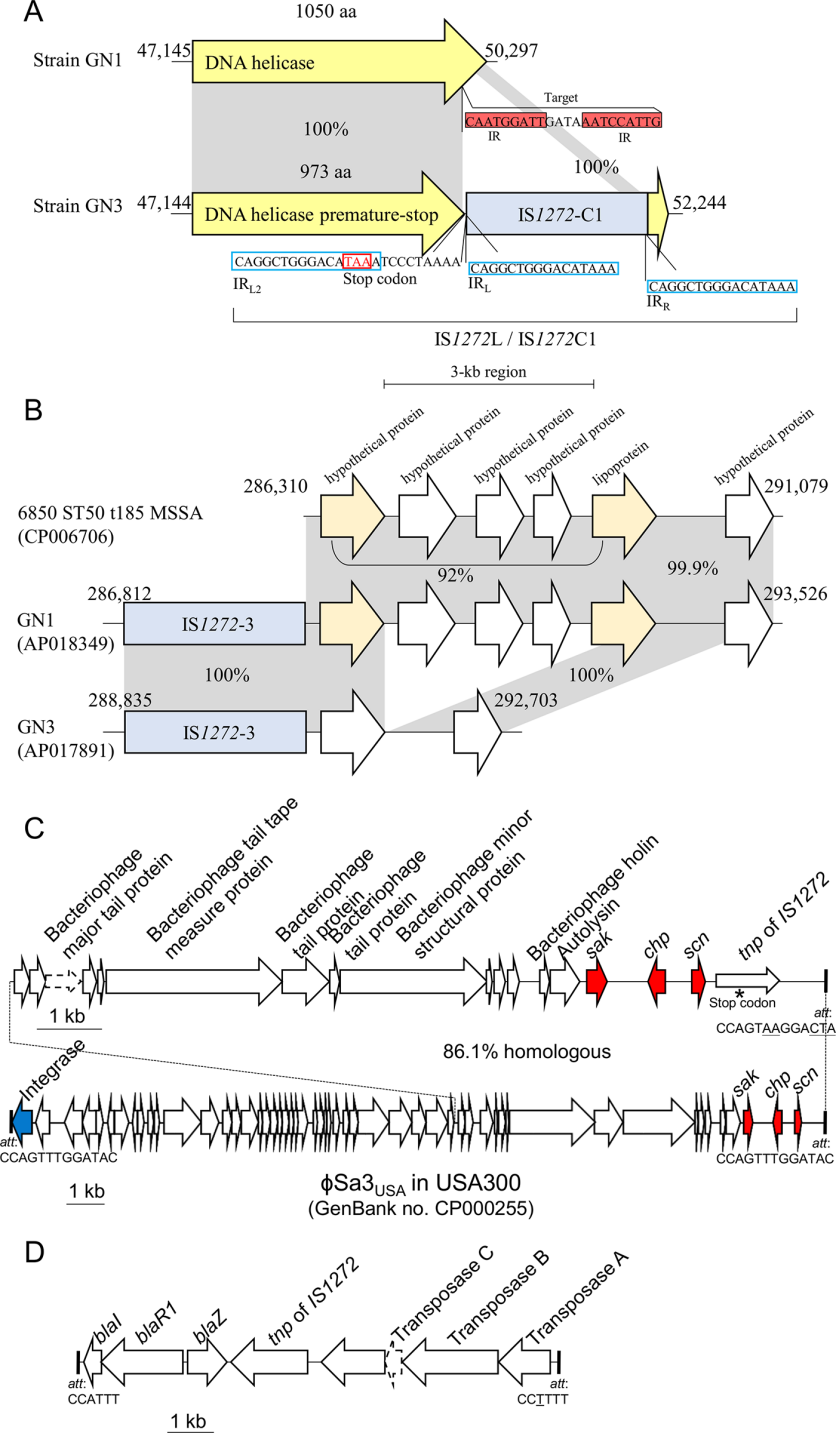
Unique genetic structures on the GN1 and GN3 genomes. (A) The GN3 genome, but not GN1 genome, had IS*1272* insertion within the DNA helicase gene, yielding newly-inserted IS*1272* (ni-IS*1272*) copy C1. The copy C1 formed a larger IS*1272* structure (IS*1272*L) with a tandem duplication of the 25-bp left side sequence including IR_L_. Due to the IS1272 C1 insertion, particularly the internal stop codon TAA which was present in IR_L2_ of IS1272L, the DNA helicase gene product was estimated to be truncated (973-aa) DNA helicase. (B) The GN3 genome had a small (3-kb) deletion close to IS*1272* (copy 3); the 3-kb region was present on the GN1 genome and also on the genome of related ST50 MSSA strain 6850. (C) The immune evasion cluster (IEC), carrying *sak* for staphylokinase (SAK), *chp* for chemotaxis inhibitory protein of *S*. *aureus* (CHIPS), and *scn* for staphylococcal complement inhibitor (SCIN), was carried by the φSa3 remnant. This structure, IEC/PR, had the *att* of φSa3; 5 out of 13 nucleotides were divergent (divergent nucleotides are underlined). Its location was not *hlb* (insertion site of φSa3). GN1 and GN3 lacked φSa3, and *hlb* was intact. The IEC/PR structure carried one IS*1272* copy (copy 7). (D) The *blaZ*,*R1*,*I-tnpA*,*B*,*C* structure encodes for penicillin resistance. This structure is flanked by short (6-bp) direct repeats. This structure also carried one IS*1272* copy (copy 11).

The location of IS*1272* may affect other genes. In the GN genomes, IS*1272* was located as follows: copy P1, at a position 66 bp downstream of the PVL F gene, *luk*_*pv*_*F*; copy 7, at a position 3 bp downstream of *scn* in IEC/PR; copy C2, at a position 15 bp downstream of *rot*; and copy 11, at a position 66 bp downstream of *blaZ* in *blaZ*-*blaR1*-*blaI* (*bla*-*tnp*). Additionally, the IS*1272* (copy C1) insertion into the DNA helicase gene resulted in a truncated DNA helicase, due to the introduction of an internal stop codon (TAA), which was present in the IR_L2_ of IS*1272* copy C1 (IS1272L) (Fig 10A). Furthermore, a small (3-kb) deletion was present close to IS*1272* (copy 3) on the GN3 genome, but not on the GN1 genome (Fig 10B).

IS*1272* (copy 7) was associated with novel genetic structure ICE/PR in the GN1 and GN3 genomes (Fig 10C). The size of IEC/PR was 21,063 bp. IEC/PR was identified as a φSa3 remnant; it corresponded to the end region of φSa3 that carries IEC, it carried φSa3 *att* (although 5 out of 13 nucleotides were divergent), and it lacked the integrase gene on the other side. Its location on each of the GN1 and GN3 genomes was far from the φSa3 insertion site (*att*) in *hlb*. IS*1272* may have played a role in the translocation of this remnant.

IS*1272* (copy 11) was inserted in the two-region array, *blaI*-*blaR1*-*blaZ* and *tnpC*-*tnpB*-*tnpA* (*bla*-*tnp*), resulting in the three-region (structure) array, *blaI*-*blaR1*-*blaZ*, IS*1272*, and *tnpC*-*tnpB*-*tnpA* with terminal direct repeats (total size, 10,294 bp) in the GN1 and GN3 genomes (Fig 10D).

### Detection of the PVL prophage with an IS*1272* insertion by PCR

The GN1 genome, but not the GN3 genome, had an IS*1272* insertion on a region downstream of the PVL F gene on the PVL prophage, as described above. When the other three GN genomes were analyzed by PCR and sequencing for the presence of this IS*1272* insertion, GN4 was negative for the IS*1272* insertion, while GN2 and GN5 were positive for it (S1 Fig).

### Expression of virulence factors

**Fig 11:**
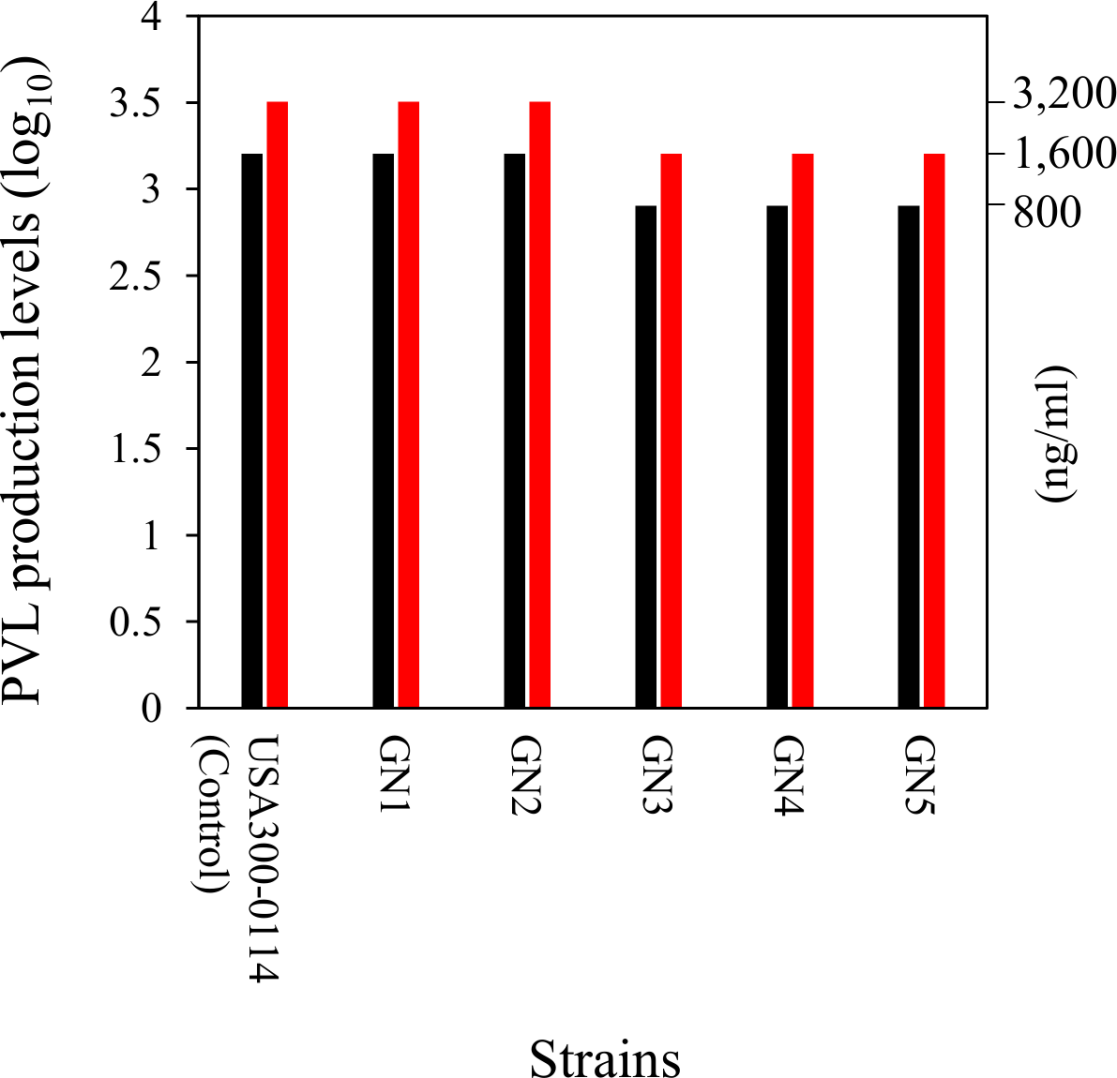
Panton-Valentine leukocidin (PVL) production levels of familial strains GN1 to GN5. Bacteria were grown in a liquid medium for 18 h with or without 5% fetal bovine serum (FBS), serial doubling dilutions of the culture supernatants were made, and the amounts of PVL in the supernatants were serologically measured. Bars (color): black, PVL production in the absence of FBS; red, PVL production in the presence of FBS. The PVL production levels of GN1 and GN2 were two-fold higher than those of GN3, GN4, and GN5. Addition of serum to the bacterial culture medium resulted in two fold-higher PVL production levels in any case. USA300-0114 was used as a PVL-positive control strain.

The PVL production levels of strains GN1 and GN2 were comparative to that of CA-MRSA USA300-0114, used as a control strain, while those of GN3, GN4, and GN5 were two-fold lower (Fig 11). Addition of serum to bacterial culture medium resulted in two fold-higher levels of PVL production in any case (Fig 11).

**Fig 12:**
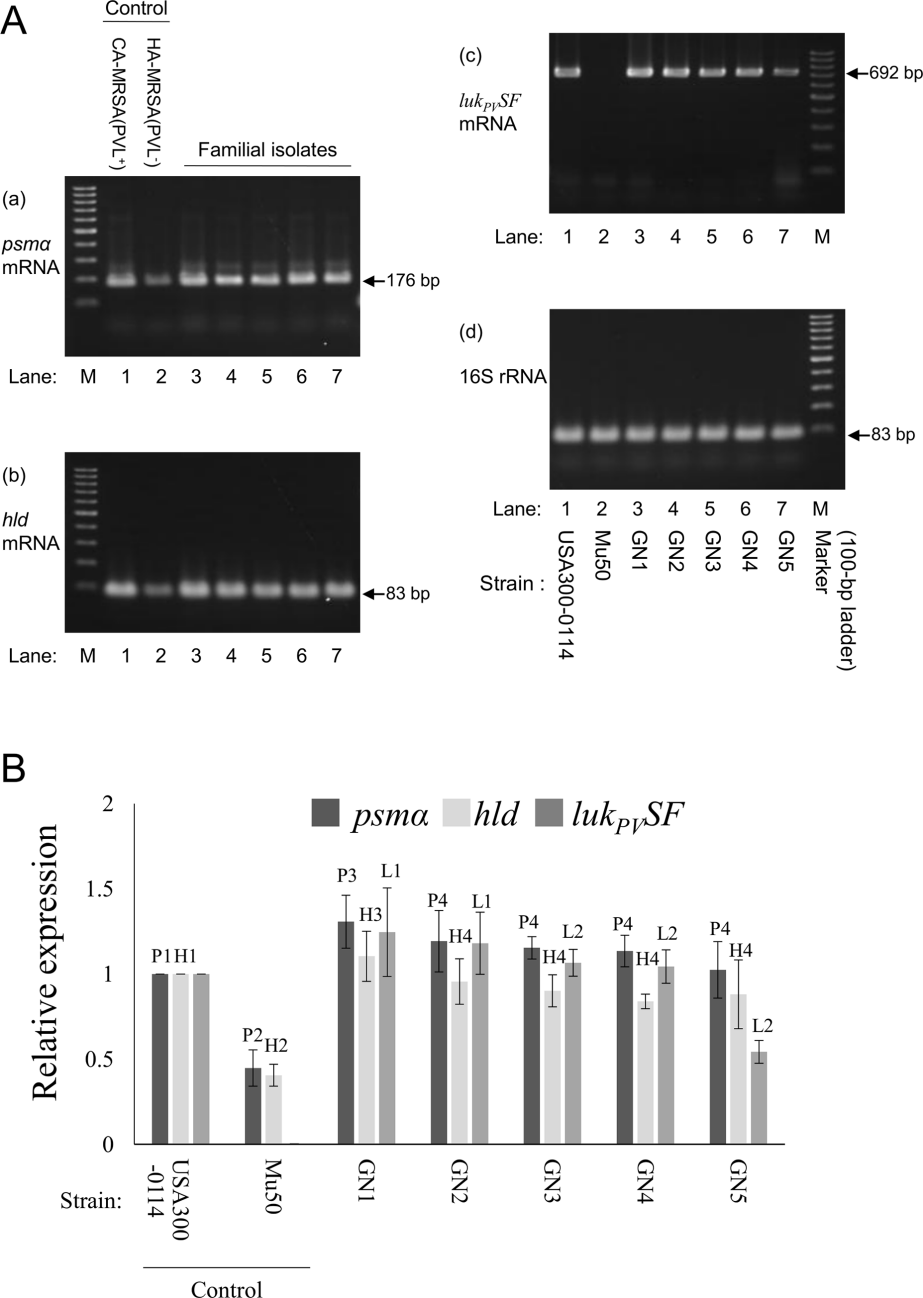
mRNA expression levels of cytolytic peptide genes (*psmα* and *hld*) and Panton-Valentine leukocidin (PVL) genes (*luk*_*PV*_*SF*) in familial strains GN1 to GN5. Bacteria were grown on sheep blood agar for 8 h, and the mRNA expression levels were examined by an RT-PCR assay. PVL-positive CA-MRSA USA300-0114 was used as a control strain which shows high expression levels for *psmα* and *hld*, and PVL-negative HA-MRSA Mu50 was used as a control strain which shows low expression levels for *psmα* and *hld*. In (A), the products in an RT-PCR assay were visualized on 2% agarose gels after electrophoresis. In (B), the expression data of each strain were normalized to those of USA300-0114. For *psmα*: P1 vs. P2, *P*<0.05; P3 vs. P2, *P*<0.05; P4 vs. P2, *P*<0.05; P3 vs. P4 (group of GN2, GN3, GN4, and GN5), *P*<0.05. For *hld*, H1 vs. H2, *P*<0.05; H3 vs. H2, *P*<0.05; H4 vs. H2, *P*<0.05; H3 vs. H4 (group of GN2, GN3, GN4, and GN5), *P*<0.05. For PVL genes: L1 (group of GN1 and GN2) vs. L2 (group of GN3, GN4, and GN5), *P*<0.05. The LVL mRNA expression level of GN5 was unexpectedly low, compared with that of other familial strains (*P*<0.05). PVL-positive CA-MRSA strain RS08 and CA-MSSA strain KT1 were also used as strong *psmα*/*hld* expression control strains, the data being comparable to that of CA-MRSA USA300-0114 (for *psmα*, 1.00, 1.09, and 0.92 for USA300-0114, RS08, and KT1, respectively; and for *hld*, 1.00, 0.85, and 0.79 for USA300-0114, RS08, and KT1, respectively); and HA-MRSA strain N315 was also used as a low *psmα*/*hld* expression control strain, the data being comparable to that of HA-MRSA Mu50 (for *psmα*, 0.45 and 0.38 for Mu50 and N315, respectively; and for *hld*, 0.41 and 0.30 for Mu50 and N315, respectively).

Regarding mRNA expression (Fig 12), all GN strains except for GN5 expressed the PVL gene at high levels that were similar to (or more than) that of CA-MRSA USA300-0114. Among GN strains, the PVL gene expression levels of GN1 and GN2 were significantly higher than those of GN3 to GN5 (*P*< 0.05). GN1 to GN5 each expressed the PSMα gene at higher levels than did HA-MRSA Mu50 (*P* < 0.05), similar to USA300-0114; notably, GN1 showed higher levels of PSMα gene expression than did the remaining GN strains (GN2 to GN5) (*P* < 0.05). Regarding the Hld gene expression, GN1 to GN5 also expressed higher levels compared with HA-MRSA Mu50 (*P* < 0.05), similar to USA300-0114, and the Hld gene expression level by GN1 was higher than that by the remaining GN strains (GN2 to GN5) (*P* < 0.05).

## Discussion

In the present study, we elucidated the complete circular genome sequences of GN, a community-associated PVL-positive *S*. *aureus* of genotype ST50/*spa*108(t185)/*agr4*, because it was associated with strong colonization and skin abscesses in a case that was part of a familial infection. ST50 *S*. *aureus* may not be a globally disseminated lineage; however, the GN *agr* type was the same as that of globally disseminated, hyper-virulent PVL-positive ST121/*agr4* MSSA [49,50]. The genome of ST50/*spa*t185 *S*. *aureus* was reported previously; this strain (6850) was isolated from a skin abscess case in Belgium, which progressed to further invasive infections such as bacteremia and osteomyelitis [51] (GenBank accession number, CP006706). Notably, the clinically important and, thus, well-characterized strain 6850 was PVL-negative and lacked phage 2, but it also carried multiple copies of IS*1272* (11 copies/genome). Having multiple IS*1272* copies may be linked with this lineage. Characteristic virulence factors for CA-MSSA GN include PVL [9,11–15,17], the stronger expressions of *psmα* and *hld* compared with HA-MRSA [12,19], collagen adhesin [52,53], and the immune evasion factors SAK, CHIPS, and SCIN [9, 27,45,54].

In 2007 [39], based on the findings obtained by PCR and sequence analyses of PVL gene regions with and without IS*1272* (GenBank accession numbers AB256036-39, 2006), we proposed the concept of stem-loop-replacing transposition. Here, to further investigate the IR-replacing transposition of IS*1272*, we attempted to elucidate the underlying mechanisms in more detail by performing a comprehensive comparison between GN genomes focusing on the status of multiple IS*1272* copies, including ni-IS*1272* and ce-IS*1272*.

**Fig 13:**
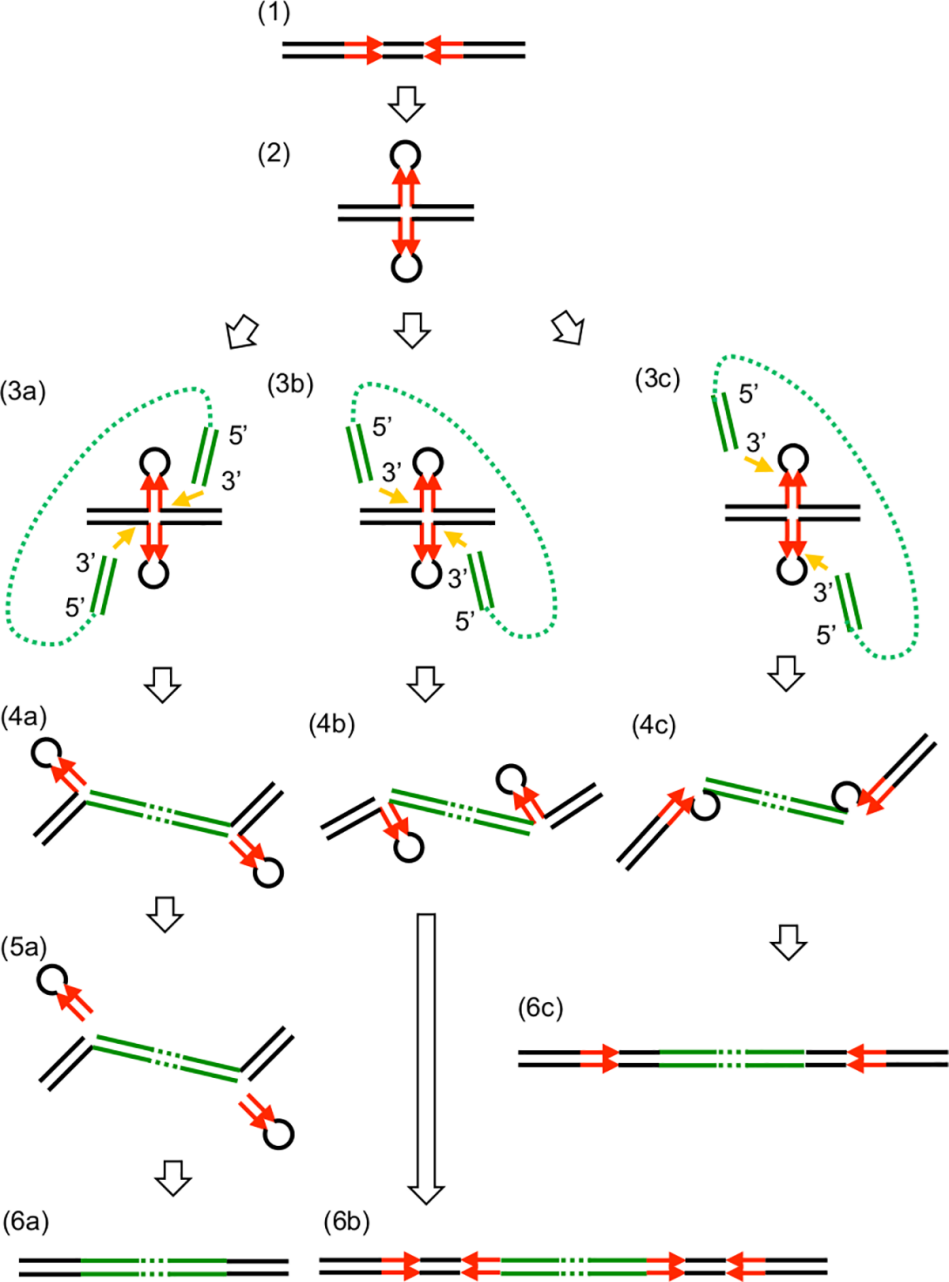
Replacement by structure-dependent transposition (RST) or stem-loop replacement: A possible model for the transposition mechanism of IS*1272*. (1) Palindromic sequences (red) are present in donor and recipient sequences. (2) Palindromic sequences form a cruciform. (3a-4a) The 3’ ends of IS*1272* (stem-loop) attack and are joined to the target DNA at the 3’ feet of two stem-loops. (5a) The stem-loops on recipient sequences are removed. (6a) IS is inserted, replacing a stem-loop sequence. (3b-4b) The 3’ ends of IS attack and are joined to the target DNA at the 5’ feet of the two stem-loop. (6b) The sequence forming the stem-loop is duplicated at both ends of IS. (3c-4c) The 3’ ends of IS attack and are joined to target DNA at the 5’ of two loops. (6c) The sequence corresponding to the two loops is duplicated at both ends of IS. Red arrows, palindromic sequences; green lines, IS DNA.

Based on the results of the present study, together with previous findings reported by others [23,28,55], we proposed a potential transposition mechanism (shown in Fig 13). Basically, in the transposition of transposable elements (TEs) catalyzed by DDE transposase, the first step is the hydrolysis of the phosphodiester backbone at each end of the TE to generate free 3’ OH ends [55]. The exposed 3’ OH ends are then joined to the target DNA in a trans-esterification reaction. Thus, neither of the 5’ ends of the TE are joined with the 3’ ends of target DNA. In cases in which the attacked site of the top strand is upstream of the site of the bottom strand, the single-stranded sequences at both sides of the TE need to be repaired. Therefore, if the 3’ ends of TE are joined to the target DNA at the 5’ foot of the stem-loop, it may result in the duplication of the whole stem-loop (Fig 13, pathway b). If the 3’ ends of the TE are joined to the target DNA at the 5’ end of the loop, it may result in the duplication of the loop only (Fig 13, pathway c). However, if the 3’ ends of the TE (IS*1272* in this case) are joined to the target DNA at the 3’ foot of the stem-loop (Fig 13 3A and 13 4A), the stem-loop cannot be replicated unless the opposite strands are cleaved. It is more likely that the two stem-loop sequences become removed by 3’ exonucleases, which results in the deletion of stem-loop sequences. We propose that this is a potential transposition mechanism for IS*1272*, and we have designated this potential mechanism as “replacement by structure-dependent transposition (RST)”, “stem-loop replacing transposition”, or “cut-paste-and-cut”. Additional evidence for this potential mechanism is currently being investigated.

In general, the transposition of ISs or any DDE-type transposons generates TSDs upon transposition [23,28]. The feature of stem-loop replacement in the IS*1272* transposition is an irreversible process, in contrast with the “canonical” transposition that can be reversed by recombinational deletions between two target site duplications [23,28,56]. The basis of the differences between “canonical” IS transposition and IS*1272* transposition is likely in their transposition mechanisms. Therefore, we may be able to use such differences to control IS*1272* without affecting other IS elements or to control other ISs without affecting IS*1272*. Saturation mutagenesis using IS*1272* is one possible direction for assessing the effect of promoting IS*1272* transposition.

It is possible that inhibitors capable of blocking the transposition of IS*1272* could help overcoming the spread of MRSA. Alternately, the promotion rather than inhibition of IS*1272* transposition may help overcome the spread of MRSA. Given that the supercoiling of genomic DNA enhances the cruciform form, intercalates that can enhance supercoiling could be candidate promoters of IS*1272* transposition, and antagonizing intercalation may be a way to inhibit the transposition of IS*1272*. Although the clinical application of these modifications is not immediate, the advance in understanding the diversity in transposition mechanisms of ISs and transposons contributes to the future control of bacterial infections.

During the preparation of this manuscript, Furi et al. [57] published a study on the transposition of two composite Tns (TnSha1 and TnSha2), which had IS*1272* as a component, and they demonstrated that these two composite Tns removed the stem-loop sequences upon transposition. However, the *tnp* of composite Tn (TnSha1) has no stop codon, resulting in a larger predicted product, and has three IS*1272* IRs in its complex structure, thereby making it difficult to elucidate the precise mechanism underlying IS*1272* transposition. Siguier et al. [28] described an IS*1182* family that included heterogeneous members (including IS*1272*), in which some members targeted palindromic sequences, but they did not provide detailed data. Thus, the present study is the first to demonstrate the precise structure, transposition, and model of IS*1272*.

**Fig 14:**
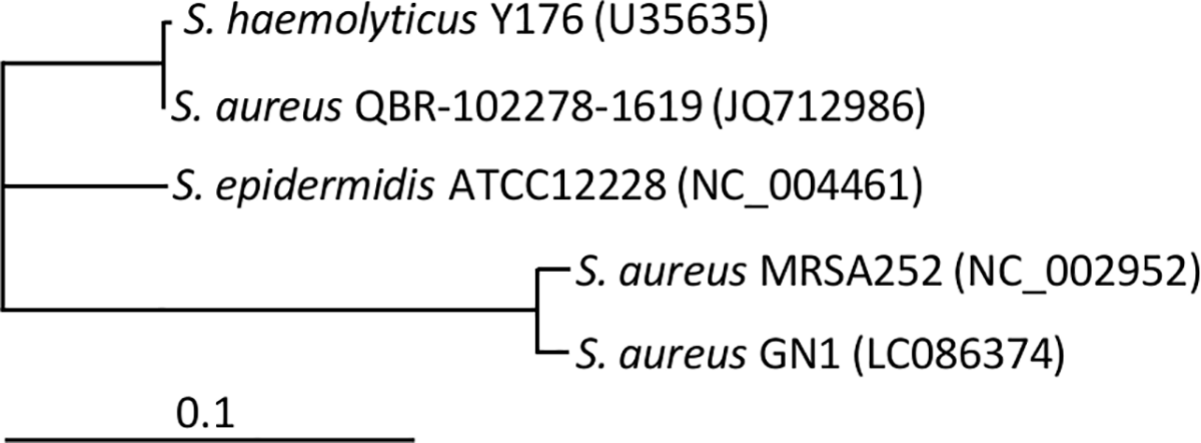
Cluster analysis of IS*1272* from *S*. *aureus*, *S*. *epidermidis*, and *S*. *haemolyticus*. GenBank accession numbers are shown in parentheses.

The cluster analysis of IS*1272* from *S*. *aureus*, *S*. *epidermidis*, and *S*. *haemolyticus* (Fig 14) indicated that our IS*1272* (GN1) showed high homology to IS*1272* from *S*. *aureus* MRSA252, but, it exhibited more divergence from the IS*1272* sequences of *S*. *epidermidis* ATCC12228 and *S*. *haemolyticus* Y176 (the original strain in which IS*1272* was initially characterized) [33]. IS*1272* from *S*. *aureus* QBR-102278-1619 is closely related to *S*. *haemolyticus* IS*1272*; however, since IS*1272* from *S*. *aureus* QBR-102278-1619 is a constituent of a composite Tn carrying *S*. *haemolyticus* gene, *fabI* [57], there is a possibility that it originated in *S*. *haemolyticus*.

Although IS*1272* was first found and characterized in *S*. *haemolyticus*, and is considered to have originally resided in *S*. *haemolyticus* [33], IS*1272* can also be an important constituent in *S*. *aureus*, for example as a multi-IS*1272* copy system. We speculate that IS*1272* has been evolving through adaptation to *S*. *aureus*.

Regarding unique genetic traits, the GN1 and GN3 genome each contained the immune evasion cluster carried by a phage 3 remnant (IEC/PR) for virulence and *blaZ*,*R1*,*I-tnpA*,*B*,*C* for drug resistance. IS*1272* was a constituent of these genetic structures. GN3, but not GN1, had a small deletion within the genome, relatively close to IS*1272*; however, the relation of IS*1272* to this deletion is not clear. IS*1272* may play a role in chromosomal rearrangements in *S*. *aureus*.

Regarding the location of IS*1272*, there were four cases of insertion that occurred very close to genes; in all four cases, IS*1272* was inserted immediately downstream of the genes.

IS*1272* is a flexible IS, but it also behaves in a strictly regulated manner. Its strict features include 16-bp IRs [33] [this study] and targets of IRs [28,39,57] [this study]. Its flexible features include variable sequences of IRs [33] [this study], variable lengths of IS*1272* [33,57] [this study], duplication of the terminal sequence including the terminal IR [this study], variable sizes and structures of *tnp* [33,57] [this study], and possibly variable modes of transposition [this study]. A large fusion transposase with a unique linker peptide region was demonstrated for the first time in the present study; however, it was a minor form.

Among the five GN familial strains, the patient strain (GN1) manifested the highest levels of PVL production and of *psmα* and *hld* expression. The importance of PSMα in SSTIs has been increasingly reported [58,59]. Although GN5 had a PVL prophage with an IS*1272* insertion, the GN5 PVL production level was comparable to those of GN3 or GN4, whose PVL prophages did not have an IS*1272* insertion. Interestingly, the GN5 PVL mRNA expression level was low compared with that of other GN strains. The background mechanism for the high virulence potential of GN1 needs to be elucidated.

In conclusion, this study is the first to present evidence for a novel IR-replacing mode of transposition and sequence data that suggests a potential stem-loop-replacing transposition mechanism for IS*1272*. We performed a comprehensive comparison of the whole-genomic sequences of familial strains of one *S*. *aureus* clone (GN) that was isolated from a five-person familial infection case. Notably, the IS*1272* transposition appeared to have occurred via an irreversible process, unlike that of the reversible “canonical” transposition of IR and Tn. IS*1272* sequences existed as a multi-IS*1272* system in the *S*. *aureus* genome, accompanying strictly regulated but also flexible structures. Basic investigation of the IS*1272* mechanism may lead to a new method of MRSA control in the future. IS*1272* was linked to IEC and drug resistance segments and was located both near a deletion and close to several genes, suggesting its possible role in chromosomal rearrangements. Although IS*1272* was originally isolated and characterized in *S*. *haemolyticus*, it plays a role in clinically important *S*. *aureus*. The present study also demonstrates that the patient strain in this familial infection case had an increased virulence potential based on community-associated virulence factors.

## Supporting information

S1 Fig PCR detection of IS*1272* insertions on the Panton-Valentine leukocidin (PVL)-prophage using GN familial strains. The applied familial strains were GN1 (patient strain) and GN2 to GN5. In the course of the previous study on the PVL S/F genes (*luk*_*PV*_*SF*), we determined the sequences of not only the entire PVL gene but also its upstream and downstream regions of clinical *S*. *aureus* isolates by PCR and sequencing, and detected an IS*1272* transposition in a region distal of the PVL F gene. PCR primers, PVL-1 and NPVL-2, were from reference [44]. Other PCR primers were designed based on the DNA sequence of a PVL prophage carried by ST30 CA-MRSA strain NN1 [37] and φPVL-Sa2_GN1_ and φPVL-Sa2_GN3_. The primers for IS*1272* were designed based on the φPVL-Sa2_GN1_ sequence, which had an IS*1272* insertion. Of three primer sets (A to C), B detects the terminal IRs of the target of IS*1272*; thus, GN1, GN2, and GN5 (in which the target was replaced by IS*1272*) produced negative results in PCR using primer set B. Six PCR primer sets (1 to 6) were used to investigate the presence of an insertion at the region located downstream of the PVL genes. The insertion of IS*1272* (size, ca. 2 kb) was checked by PCR product sizes and PCR product sequences. GN1, GN2, and GN5 had a ca. 2-kb insertion (corresponding IS*1272*), while the parent's strains (GN3 and GN4) did not.
(TIF)

S2 Fig Structures of PVL-coverting prophages, φPVL-Sa2_GN1_ and φPVL-Sa2_GN3_. φPVL-Sa2_GN1_ (of patient strain GN1) had IS*1272* insertion (IS*1272* copy P1) in a region distal to the PVL F gene, while φPVL-Sa2_GN3_ (of a parent/female strain) had no IS*1272* insertion. φPVL-Sa2_GN1_ and φPVL-Sa2_GN3_ were highly homologous to φPVL-Sa2 of JCSC7401 (ST80 MRSA).
(TIF)

S3 Fig Database searches for the target/IS*1272* sequence sets for IS*1272*. Color: red, target inverted repeats (IRs); yellow, IS*1272* with terminal IRs; blue, the same DNA sequence region between the genomes carrying the targets or IS*1272* insertion. Target/transposition set P1 represents an example obtained by comparison between the GN1 and GN3 genomes.
(TIF)
