# Supplementary figures and images for: Genomic comparison between Staphylococcus aureus GN strains clinically isolated from a familial infection case: IS1272 transposition through a novel inverted repeat-replacing mechanism
Source: PLoS One. 2017 Nov 8;12(11):e0187288. doi: 10.1371/journal.pone.0187288 (PMC5678879; doi:10.1371/journal.pone.0187288)

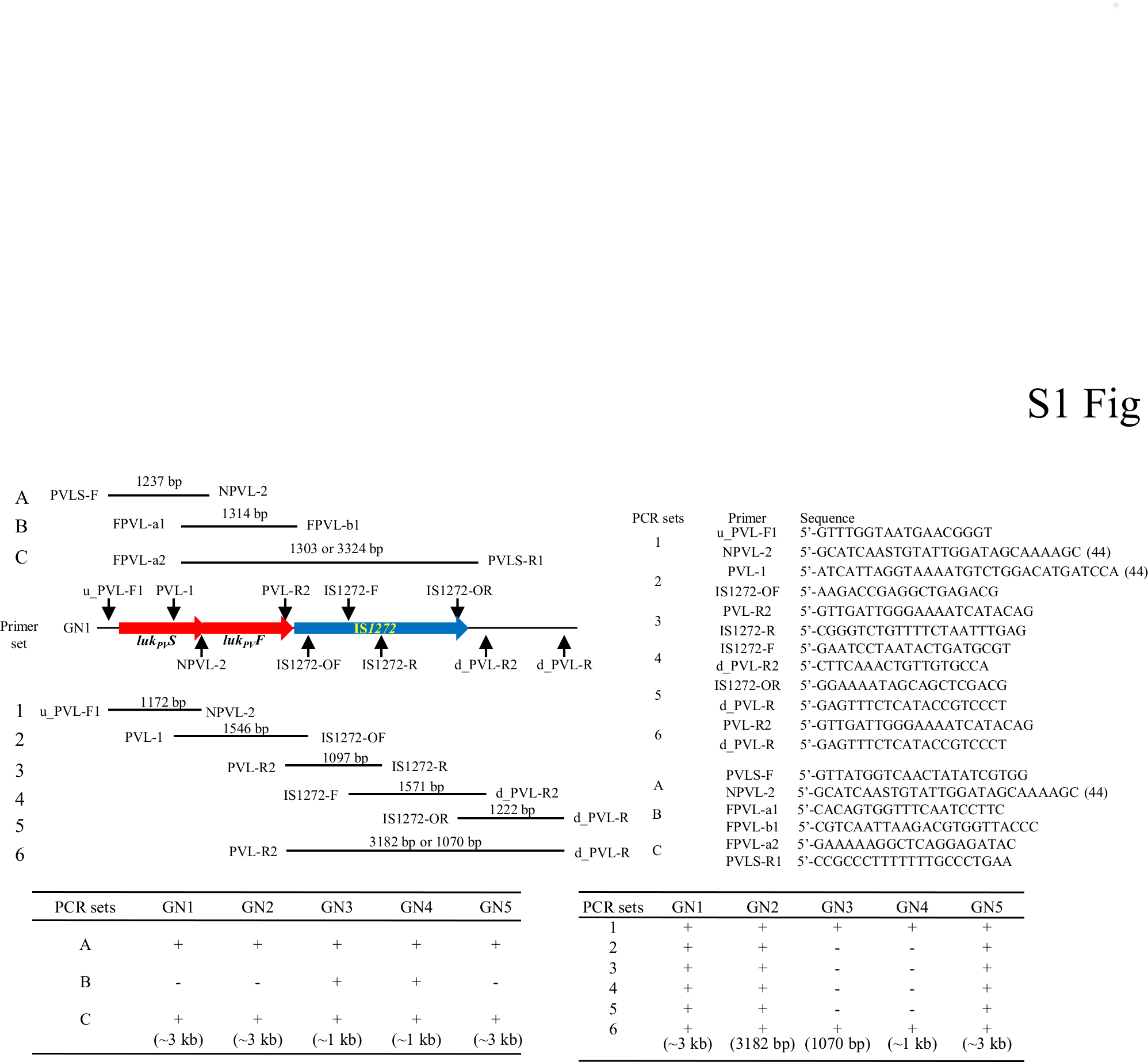

Supplement: S1 Fig — The applied familial strains were GN1 (patient strain) and GN2 to GN5. In the course of the previous study on the PVL S/F genes (lukPVSF), we determined the sequences of not only the entire PVL gene but also its upstream and downstream regions of clinical S. aureus isolates by PCR and sequencing, and detected an IS1272 transposition in a region distal of the PVL F gene. PCR primers, PVL-1 and NPVL-2, were from reference [44]. Other PCR primers were designed based on the DNA sequence of a PVL prophage carried by ST30 CA-MRSA strain NN1 [37] and φPVL-Sa2GN1 and φPVL-Sa2GN3. The primers for IS1272 were designed based on the φPVL-Sa2GN1 sequence, which had an IS1272 insertion. Of three primer sets (A to C), B detects the terminal IRs of the target of IS1272; thus, GN1, GN2, and GN5 (in which the target was replaced by IS1272) produced negative results in PCR using primer set B. Six PCR primer sets (1 to 6) were used to investigate the presence of an insertion at the region located downstream of the PVL genes. The insertion of IS1272 (size, ca. 2 kb) was checked by PCR product sizes and PCR product sequences. GN1, GN2, and GN5 had a ca. 2-kb insertion (corresponding IS1272), while the parent's strains (GN3 and GN4) did not. (TIF) [file pone.0187288.s001.tif]

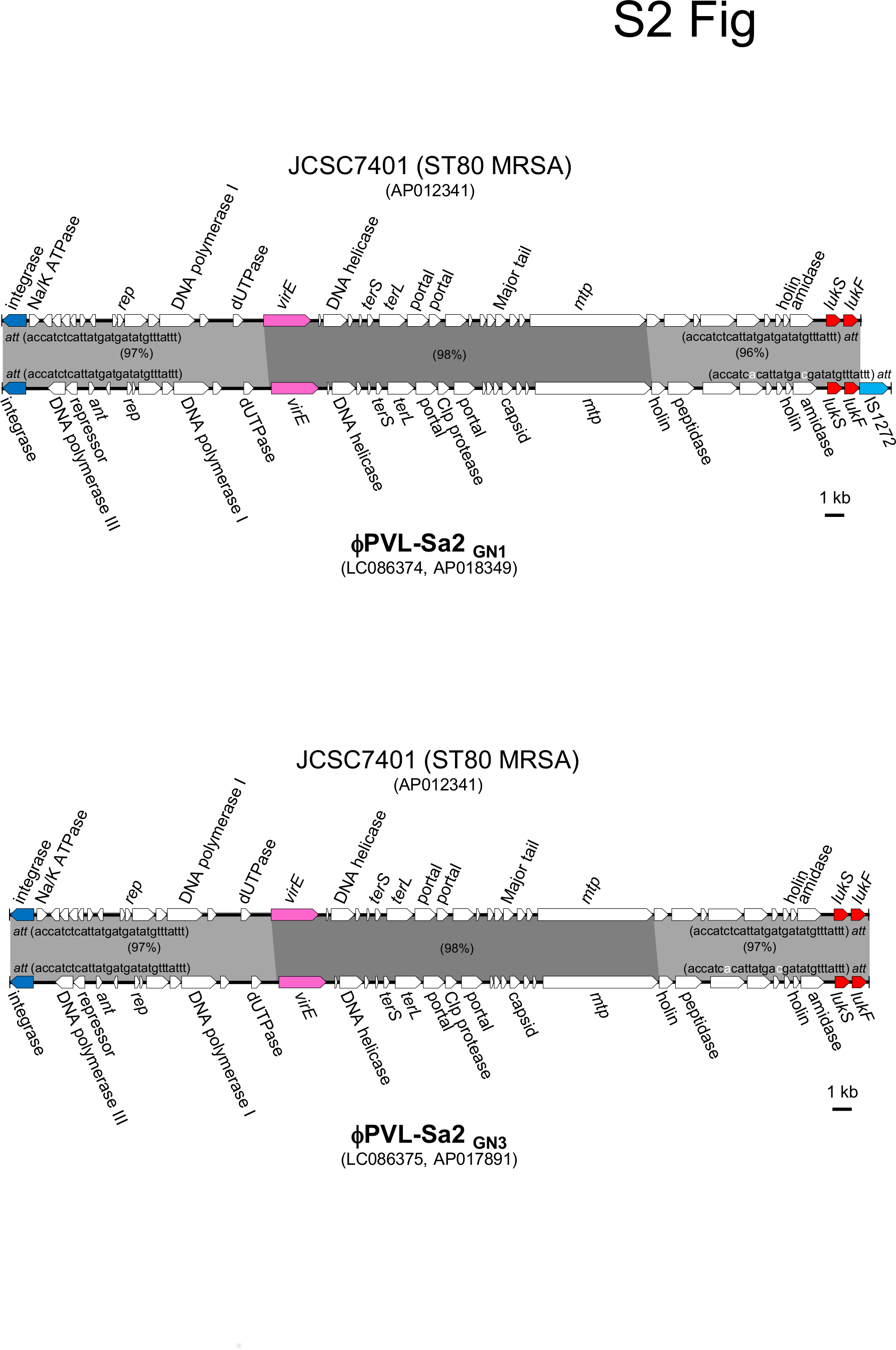

Supplement: S2 Fig — φPVL-Sa2GN1 (of patient strain GN1) had IS1272 insertion (IS1272 copy P1) in a region distal to the PVL F gene, while φPVL-Sa2GN3 (of a parent/female strain) had no IS1272 insertion. φPVL-Sa2GN1 and φPVL-Sa2GN3 were highly homologous to φPVL-Sa2 of JCSC7401 (ST80 MRSA). (TIF) [file pone.0187288.s002.tif]

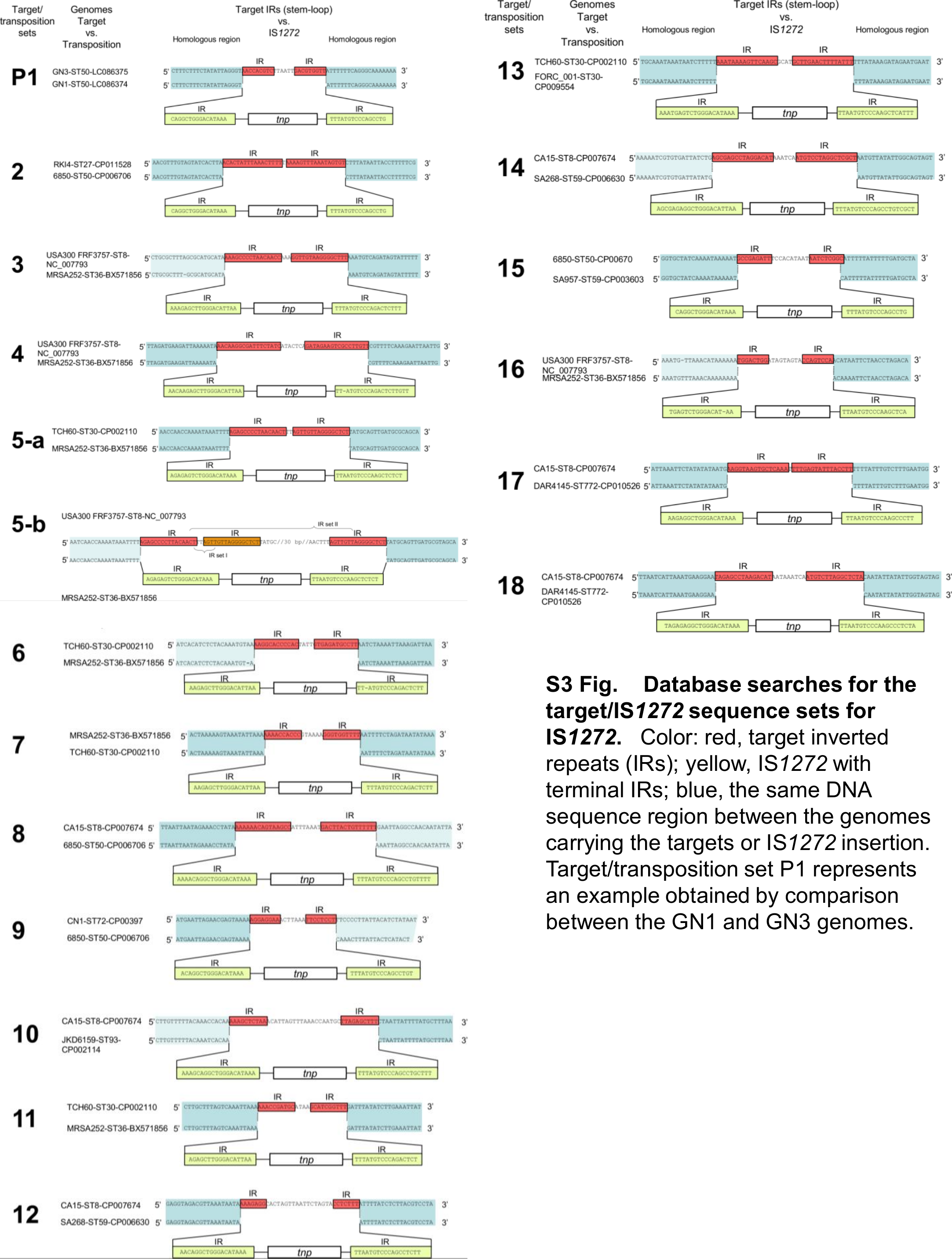

Supplement: S3 Fig — Color: red, target inverted repeats (IRs); yellow, IS1272 with terminal IRs; blue, the same DNA sequence region between the genomes carrying the targets or IS1272 insertion. Target/transposition set P1 represents an example obtained by comparison between the GN1 and GN3 genomes. (TIF) [file pone.0187288.s003.tif]
